# PI3K/AKT/mTOR signaling at the immune checkpoint interface in lung cancer: molecular mechanisms, Tumor–Immune remodeling, therapeutic resistance, and emerging treatment strategies

**DOI:** 10.3389/fcell.2026.1952310

**Published:** 2026-09-25

**Authors:** Hongliang Jin, Fan Yang, Shi Zong, Wenli Sun

**Affiliations:** Department of Thoracic Surgery, Shenyang Tenth People’s Hospital (Shenyang Chest Hospital), Shenyang, Liaoning, China

**Keywords:** combination immunotherapy, immune blockade, immune checkpoint inhibitors, immune evasion, PD-1, PD-L1, PI3K/akt/mTOR signaling

## Abstract

Immune checkpoint inhibitors targeting the PD-1/PD-L1 axis have substantially improved the treatment of lung cancer, yet durable responses remain limited by primary and acquired resistance. Increasing evidence identifies the PI3K/AKT/mTOR pathway as a central link between oncogenic signaling, metabolic adaptation, and tumor immune suppression. Genetic and molecular alterations, including PIK3CA activation, PTEN loss, and aberrant EGFR or ALK signaling, can activate this pathway and regulate PD-L1 expression at transcriptional, translational, and post-translational levels. Conversely, tumor-intrinsic PD-L1 signaling can reinforce PI3K/AKT/mTOR activity, promoting proliferation, survival, glycolysis, epithelial–mesenchymal transition, and resistance to therapy. This reciprocal interaction establishes a feedforward circuit that supports lung cancer progression and immune escape. The pathway also reshapes the tumor microenvironment by promoting regulatory T-cell expansion, M2-like macrophage polarization, myeloid-cell recruitment, CD8^+^ T-cell dysfunction, and resistance to immune checkpoint blockade. This review critically examines the bidirectional crosstalk between PI3K/AKT/mTOR and PD-1/PD-L1 signaling in lung cancer and summarizes the molecular regulators that connect these pathways. We further discuss therapeutic strategies designed to disrupt this network, including PI3K, AKT, and mTOR inhibitors; targeted therapies; chemotherapy and radiotherapy combinations; antiangiogenic approaches; nanomedicine; extracellular-vesicle-based delivery; cell-based interventions; and natural compounds. Although preclinical evidence supports combining pathway inhibition with immune checkpoint blockade, clinical translation is challenged by pathway complexity, context-dependent regulation of PD-L1, systemic toxicity, and the absence of validated predictive biomarkers. Biomarker-guided patient selection, optimized dosing and sequencing, and selective targeting of tumor- or immune-cell-specific pathway components will therefore be essential for converting this mechanistic rationale into durable clinical benefit.

## Graphical Abstract

**Figure:**
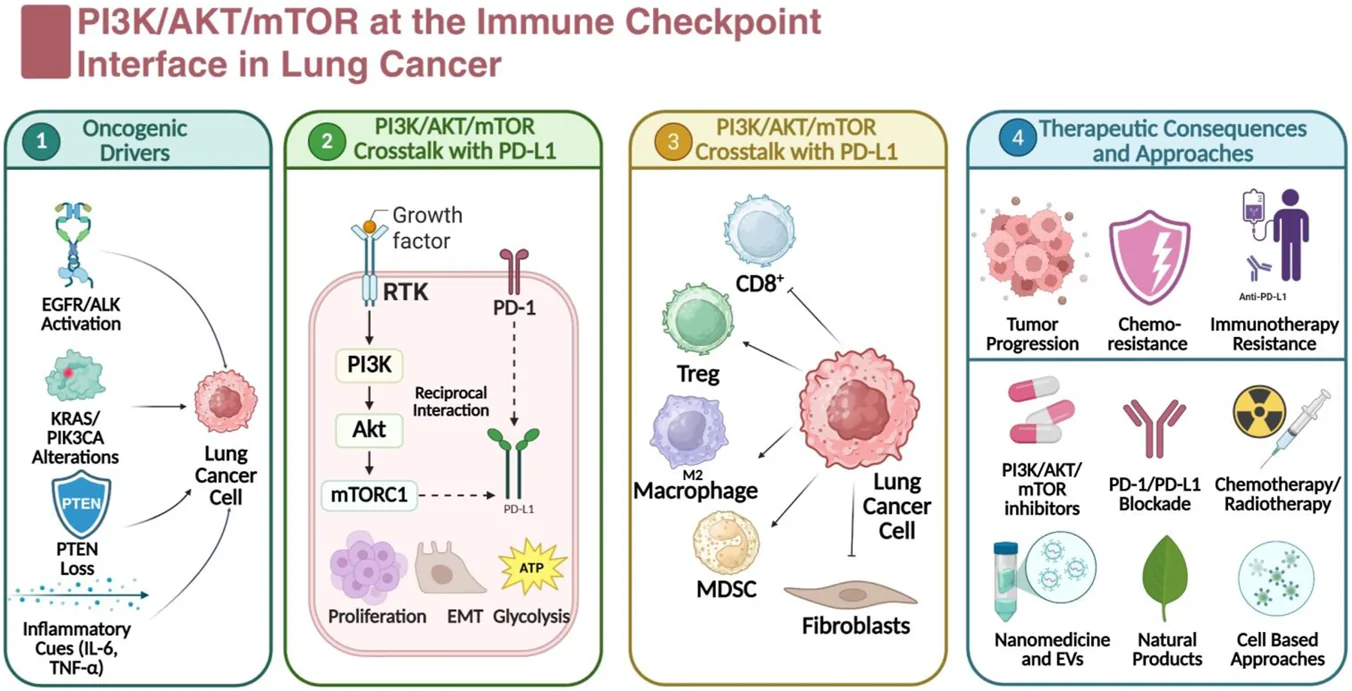

## 1 Introduction

Lung cancer was the most commonly diagnosed malignancy worldwide in 2022, with approximately 2.5 million new cases, representing 12.4% of all newly diagnosed cancers (Bray et al., 2024), despite existing treatments such as surgery, chemotherapy, radiotherapy, and targeted therapy (Li et al., 2023). Chemotherapy and radiotherapy continue to serve as major components of lung cancer management, whereas immunotherapy has emerged as the newest major systemic approach by stimulating or restoring the ability of the immune system to recognize and eliminate malignant cells (Lahiri et al., 2023). Therapeutic targeting of the programmed death ligand 1 (PD-L1) pathway represents one of the most important advances in the recent treatment of lung cancer. This strategy interferes with the programmed death 1/programmed death ligand 1 (PD-1/PD-L1) signaling axis, which is frequently exploited by tumor cells to escape immune recognition and destruction (Wang et al., 2023). Under physiological conditions, the PD-1/PD-L1 pathway regulates peripheral T-cell responses, maintains immune tolerance, limits excessive immune activation, and protects normal tissues from autoimmune injury (Lin et al., 2024). However, lung cancer cells can aberrantly overexpress PD-L1, enabling its interaction with PD-1 receptors on activated T cells and consequently suppressing antitumor immune responses. Immune checkpoint inhibitors targeting PD-1 or PD-L1, including pembrolizumab, nivolumab, and atezolizumab, disrupt this inhibitory interaction and restore the capacity of T cells to recognize and attack tumor cells. These agents have produced substantial improvements in survival and quality of life, particularly among patients whose tumors exhibit high PD-L1 expression. Consequently, PD-1/PD-L1 inhibitors have become a standard component of treatment for many patients with lung cancer, either as monotherapy or in combination with chemotherapy and other therapeutic approaches (Qu et al., 2021). The phosphatidylinositol 3-kinase/protein kinase B/mechanistic target of rapamycin (PI3K/AKT/mTOR) signaling pathway is another central regulator of lung cancer initiation, progression, and therapeutic resistance (Mohanty et al., 2024). This intracellular network regulates cell proliferation, survival, growth, metabolism, and adaptation to stressful conditions. Its persistent activation, caused by alterations such as PIK3CA mutations, AKT amplification, or PTEN loss, promotes oncogenesis and supports tumor-cell survival under conditions such as hypoxia (Rascio et al., 2021). Aberrant PI3K/AKT/mTOR activity also facilitates angiogenesis, invasion, and metastasis while contributing to resistance to chemotherapy, radiotherapy, and targeted agents, including EGFR inhibitors (Qiang et al., 2025). For these reasons, inhibition of PI3K/AKT/mTOR signaling has been proposed as a personalized therapeutic approach in non-small-cell lung cancer (NSCLC). Blocking this pathway may directly restrict tumor growth, restore sensitivity to upstream targeted therapies, and improve treatment efficacy when guided by molecular profiling (Papadimitrakopoulou, 2012). Because PI3K/AKT signaling regulates both malignant-cell behavior and the immunosuppressive tumor microenvironment, its inhibition may simultaneously suppress oncogenic activity and strengthen antitumor immunity. The pathway therefore remains a clinically relevant target for integration with contemporary immunotherapeutic regimens (Caforio et al., 2021). PI3K/AKT/mTOR signaling also intersects with PD-1, PD-L1, CTLA-4, and CD28 pathways that control T-cell activation, proliferation, metabolism, and effector function. This interaction provides a rationale for combining PI3K or mTOR inhibitors with immune checkpoint blockade to restore T-cell activity and metabolic fitness while limiting immune suppression (Wang S. et al., 2024). Moreover, acquired resistance to PD-1/PD-L1 inhibitors in lung cancer is associated with heterogeneous genomic and immune alterations, including defects in antigen presentation and interferon signaling, changes involving mTOR, reduced HLA class I expression, and decreased T-cell infiltration. These findings support individualized strategies that combine genomic targeting with immune modulation to recover responsiveness to checkpoint inhibition (Ricciuti et al., 2024). The relationship between PD-1/PD-L1 signaling and tumor biology is nevertheless context-dependent. PD-1 and PD-L1 may also be expressed intrinsically by lung cancer cells, and tumor-cell-autonomous PD-1/PD-L1 signaling has been reported to inhibit cellular growth through suppression of AKT and ERK1/2 activity (Wang et al., 2020b). This observation suggests that checkpoint signaling may exert effects beyond immune-cell inhibition and should also be considered as a tumor-intrinsic regulatory mechanism. This review critically examines the bidirectional interaction between PI3K/AKT/mTOR signaling and the PD-1/PD-L1 axis in lung cancer, with particular attention to immune escape and therapeutic resistance. We summarize how PI3K/AKT/mTOR regulates PD-L1 expression and immune-cell function, as well as how tumor-intrinsic PD-L1 can reciprocally influence this signaling pathway. We also discuss emerging pharmacological, genetic, targeted, radiation-based, nanotechnology-based, extracellular-vesicle-based, and cell-based approaches designed to simultaneously disrupt these interconnected pathways and improve the efficacy of immune checkpoint blockade.

## 2 PD-L1 regulation by PI3K/AKT/mTOR pathway

PD-L1 expression in NSCLC is partly controlled by EGFR-dependent PI3K/AKT signaling. EGF increased PI3K/AKT activity and PD-L1 expression, whereas LY294002 reduced both basal and EGF-induced PD-L1, indicating that this pathway contributes to, but does not exclusively determine, PD-L1 regulation (Okita et al., 2017). Similarly, oncogenic AKT–mTOR activation in EGFR-, KRAS-, and NNK-driven models correlated with increased PD-L1. PI3K/AKT/mTOR blockade, particularly RAPTOR-mediated mTORC1 inhibition, reduced PD-L1, which was regulated mainly at the translational and lysosomal-turnover levels. IFN-γ- and EGF-induced PD-L1 required mTOR rather than MEK–ERK signaling, and PD-L1 strongly correlated with pS6 in human lung tumors. In KRAS-driven mice, rapamycin combined with anti-PD-1 produced the greatest tumor reduction and increased CD3^+^ infiltration and the CD3^+^/FoxP3^+^ ratio (Lastwika et al., 2016). IFN-γ simultaneously activates growth-inhibitory JAK2/STAT1 and compensatory PI3K/AKT signaling, with the latter promoting PD-L1 expression and limiting IFN-γ-mediated antitumor effects. PI3K inhibition therefore reduced PD-L1 and enhanced responsiveness to IFN-γ, suggesting reciprocal regulation between PI3K/AKT and STAT1-dependent transcription (Gao et al., 2018). In EGFR-mutant NSCLC, this regulation also involved AKT-dependent STAT3 phosphorylation and nuclear translocation. EGFR, AKT, or STAT3 inhibition decreased PD-L1, including in gefitinib-resistant T790M cells, indicating a potential strategy for overcoming EGFR-TKI resistance (Abdelhamed et al., 2016). Consistent with this possibility, EGFR-mutant tumors with high PD-L1 expression or tumor mutational burden frequently harbored PI3K-pathway alterations, particularly PIK3CA mutations and PTEN loss. These tumors showed greater mutation burdens and distinct co-mutation patterns, suggesting that PI3K activation may contribute to primary EGFR-TKI resistance and provide a rationale for combining PI3K and EGFR inhibition (Ding et al., 2024). However, the direction of PD-L1 regulation by mTORC1 is not uniform across experimental systems. Whereas Lastwika et al. showed that pharmacological inhibition of PI3K, AKT, or mTOR and genetic depletion of RAPTOR reduced PD-L1 expression, Deng et al. reported the opposite effect, with inhibition of mTORC1/p70S6K increasing total and cell-surface PD-L1 in several cancer cell lines. These apparently contradictory findings may reflect distinct layers of PD-L1 regulation rather than a simple binary relationship between mTORC1 activity and PD-L1 abundance. Lastwika et al. demonstrated that oncogenic and IFN-γ-induced PD-L1 protein expression depends on active AKT–mTOR signaling and emphasized that mTOR activation is necessary but not sufficient for PD-L1 expression, because availability of PD-L1 transcripts and the relative contribution of transcriptional versus translational regulation vary among cellular contexts. In contrast, Deng et al. found that mTORC1 inhibition produced little or no increase in PD-L1 mRNA in most tested NSCLC cells, indicating that the paradoxical increase in PD-L1 occurred predominantly at the post-translational level. Mechanistically, mTORC1/p70S6K inhibition accelerated proteasomal degradation of the E3 ubiquitin ligase β-TrCP, thereby reducing PD-L1 degradation and increasing PD-L1 protein stability. These observations suggest that the net effect of mTORC1 inhibition may depend on the balance between PD-L1 synthesis and turnover in a given tumor cell. When PD-L1 abundance is strongly dependent on mTORC1-supported translation, pathway inhibition may reduce PD-L1; conversely, where an existing PD-L1 protein pool is substantially controlled by β-TrCP-dependent degradation, inhibition of mTORC1/p70S6K may stabilize PD-L1 and increase its surface abundance. Basal PD-L1 and PD-L1 transcript levels, oncogenic and signaling context, and cell-specific differences in protein-turnover machinery may therefore influence the direction of the response. Methodological differences may also contribute, as Deng et al. noted differences in the PD-L1 antibodies used and in the proportion of cell lines with detectable basal PD-L1 between the two studies. Temporal effects alone are unlikely to fully explain the discrepancy because Deng et al. observed increased PD-L1 as early as 4 h after mTOR inhibition, with the increase persisting at later time points. Thus, mTORC1 should be viewed as a context-dependent regulator of PD-L1 homeostasis whose inhibition can shift the relative contributions of protein synthesis and degradation in opposite directions (Lastwika et al., 2016; Deng et al., 2019). PI3K inhibition also produced divergent transcriptional and protein-level effects in IFN-β-treated lung cancer cells: LY294002 increased PD-L1 mRNA but decreased surface protein. Thus, PI3K signaling may suppress PD-L1 transcription while supporting its translation, trafficking, or stability, highlighting multilayered and context-dependent regulation of PD-L1 by the PI3K/AKT/mTOR pathway (Figure 1) (Morimoto et al., 2018).

**FIGURE 1:**
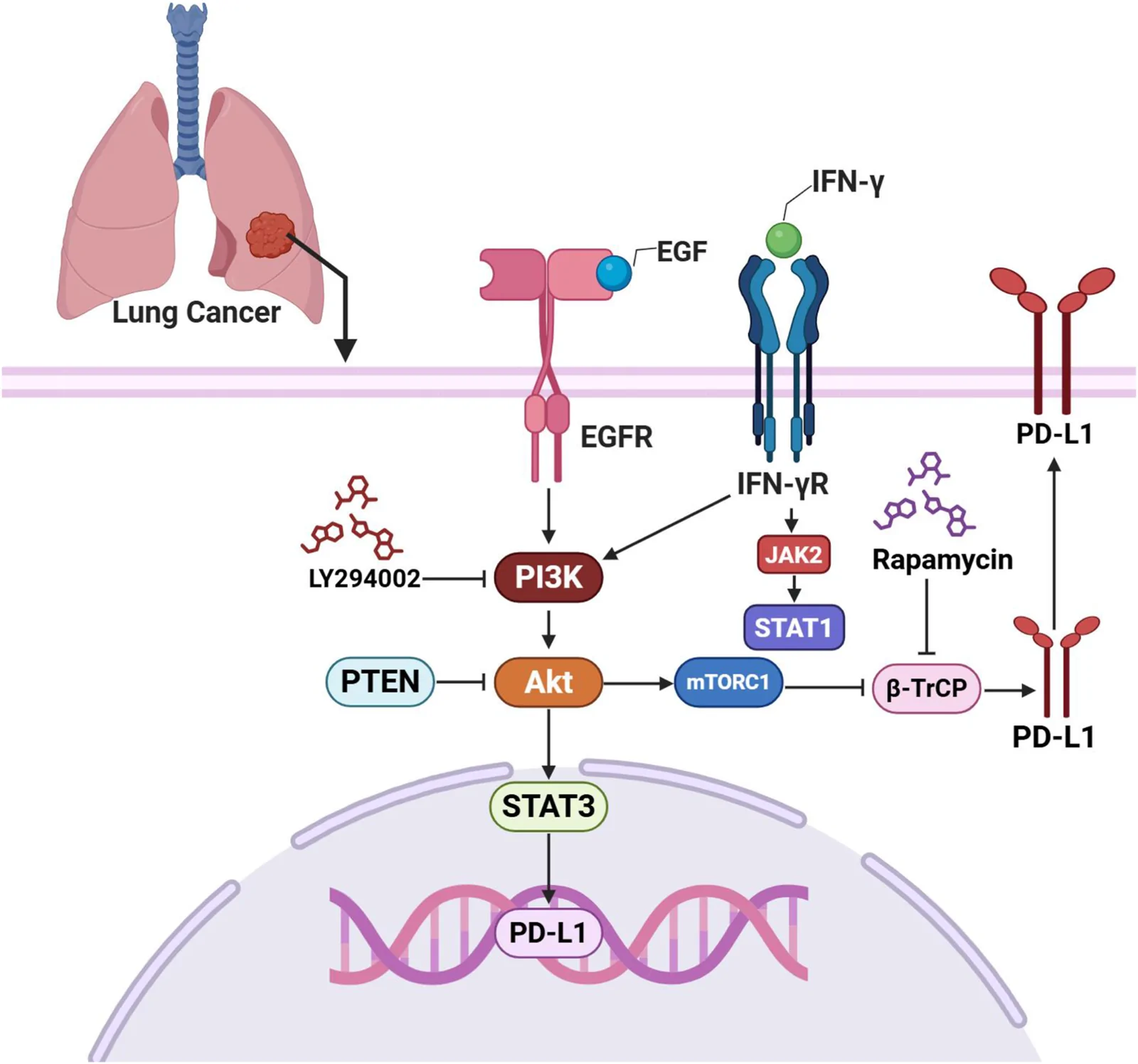
Schematic overview of EGFR- and IFN-γ–dependent regulation of PD-L1 through PI3K/AKT/mTOR signaling in NSCLC. EGF binding to EGFR activates PI3K/AKT signaling, promoting PD-L1 expression through AKT–STAT3-dependent transcription and mTORC1-dependent regulation of PD-L1 protein abundance. IFN-γ binds its canonical receptor, IFN-γR (IFNGR1/2), rather than EGFR, and activates JAK2/STAT1 signaling while also engaging PI3K/AKT signaling, thereby contributing to PD-L1 regulation. LY294002 inhibits PI3K, whereas PTEN negatively regulates AKT signaling. mTORC1-dependent control of PD-L1 is context dependent: active mTORC1 can support PD-L1 synthesis, whereas mTORC1/p70S6K inhibition by rapamycin may decrease β-TrCP abundance, reduce PD-L1 degradation, and consequently increase PD-L1 protein stability and cell-surface expression.

### 2.1 Cellular mechanisms

#### 2.1.1 Macrophage functions

Macrophage-intrinsic Ubc13 (UBE2N) restrains immunosuppressive TAM reprogramming in lung cancer. Myeloid-specific Ubc13 deletion enhanced primary tumor growth and lung metastasis, impaired CD8^+^ T-cell proliferation and effector differentiation, increased MDSC recruitment, and upregulated PD-L1 and Arg1 in TAMs. Mechanistically, Ubc13 promoted K63-linked Akt polyubiquitination and limited Akt activation, whereas Ubc13 loss increased Akt phosphorylation, Cd274 transcription, and PD-L1 expression in macrophages exposed to IL-4 or tumor-derived signals. Ubc13 deficiency therefore shifts macrophages toward a protumoral phenotype that suppresses antitumor T-cell immunity (Figure 2) (Sun et al., 2025).

**FIGURE 2:**
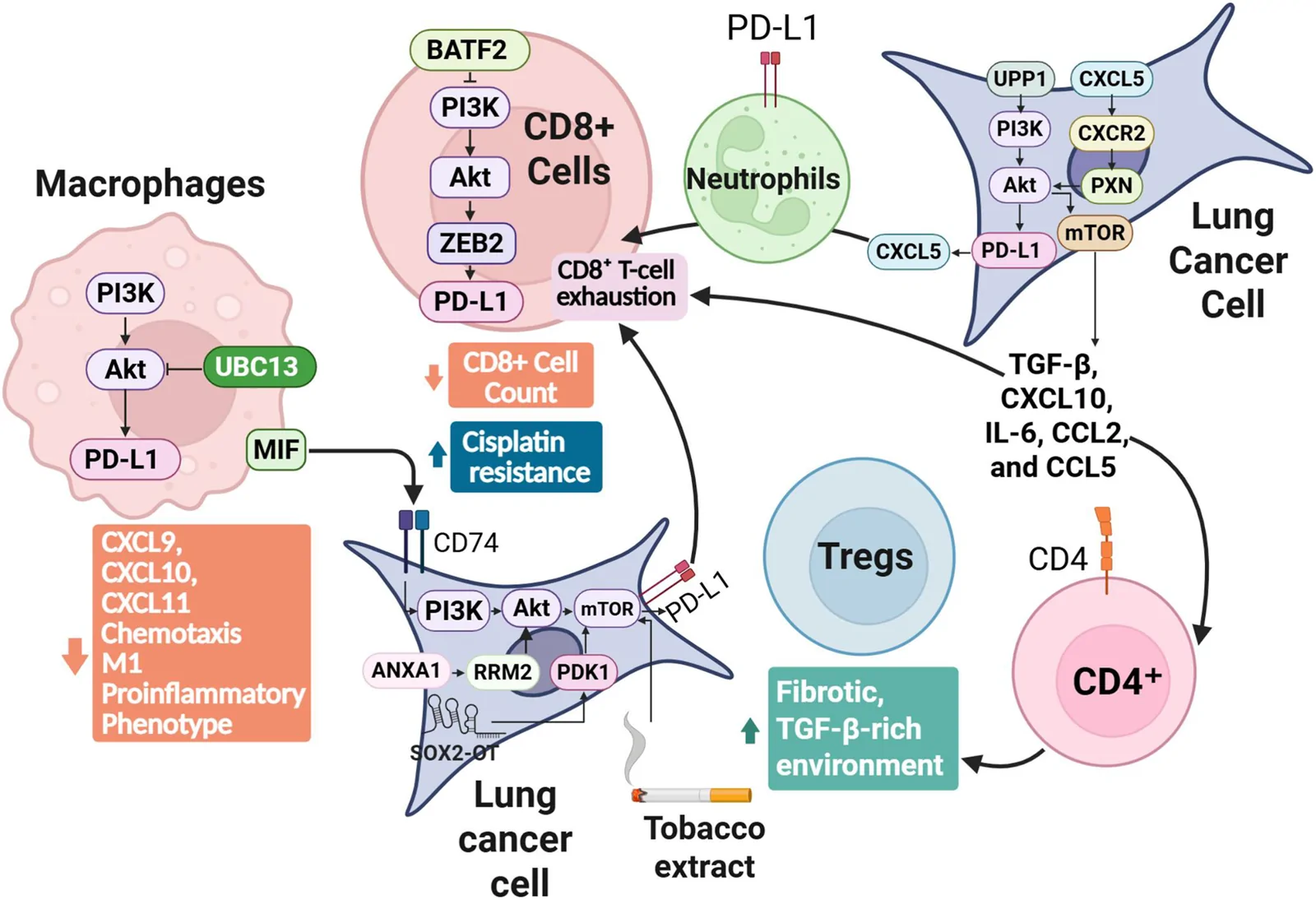
Intercellular crosstalk driving immune evasion and therapy resistance in lung cancer. Diagram depicting dynamic interactions among tumor cells, macrophages, neutrophils, CD8^+^ T cells, CD4^+^ T cells, and Tregs that collectively establish an immunosuppressive tumor microenvironment. Tumor cell–intrinsic activation of PI3K/AKT/mTOR signaling induces PD-L1 expression and shapes paracrine outputs, including CXCL5, TGF-β, IL-6, and other chemokines, which recruit and reprogram immune cells. CXCL5-mediated neutrophil infiltration and PD-L1^+^ neutrophils promote CD8^+^ T-cell exhaustion, while macrophage-derived MIF engages CD74 on tumor cells to further enhance PD-L1 expression and suppress cytotoxic immunity. Loss of macrophage Ubc13 skews TAMs toward an immunosuppressive phenotype, reinforcing PD-L1–dependent T-cell dysfunction. Concurrently, tumor-derived cytokines drive CD4^+^ T-cell differentiation into FOXP3^+^ Tregs, generating a TGF-β–rich, fibrotic niche that limits effective antitumor responses and contributes to chemoresistance and immune escape.

#### 2.1.2 CD8^+^ cells

PD-L1 contributes to cisplatin resistance in NSCLC partly by suppressing CD8^+^ T-cell activity. In 92 patients receiving neoadjuvant chemotherapy, high PD-L1 expression was associated with advanced TNM stage, poor treatment response, and reduced overall survival, while chemotherapy-induced increases in PD-L1 predicted resistance. Cisplatin also dose-dependently increased PD-L1 in NSCLC cell lines and patient-derived xenografts. PD-L1 silencing or PI3K/AKT inhibition restored cisplatin sensitivity, increased apoptosis, and was consistent with the inverse association between tumor PD-L1 and CD8^+^ T-cell infiltration in patient biopsies (Zhang et al., 2016). CXCL5 similarly promotes immune escape through a CXCR2/PXN/AKT/PD-L1 circuit. CXCL5 was elevated in lung tumors and associated with poor prognosis. It activated CXCR2, paxillin, and AKT in tumor cells, increasing PD-L1 and reinforcing CXCL5 expression through positive feedback. CXCL5 also recruited neutrophils, while neutrophil-derived GM-CSF further activated PXN/AKT signaling. The resulting PD-L1^+^ neutrophils impaired CD8^+^ T-cell chemotaxis and cytotoxicity. Dual CXCL5 and PD-L1 blockade suppressed tumor growth and restored T-cell infiltration *in vivo* without apparent toxicity (Sun et al., 2024). Conversely, BATF2 limits NSCLC progression by suppressing the PI3K/AKT/ZEB2/PD-L1 pathway. BATF2 expression negatively correlated with PD-L1 in patient plasma and tumor tissues, whereas BATF2 loss increased PI3K/AKT activation, nuclear ZEB2, and PD-L1 transcription. In mice, BATF2 deficiency increased PD-L1 and ZEB2, while BATF2 overexpression enhanced dendritic-cell, NK-cell, and cytotoxic CD8^+^ T-cell infiltration (Figure 2) (Liu et al., 2023).

#### 2.1.3 Regulatory T Cells

PTEN loss activates AKT/mTOR and JAK/STAT3 signaling, generating an immunosuppressive microenvironment that promotes resistance to PD-1 blockade. PTEN-deficient tumor cells increased secretion of TGF-β, CXCL10, IL-6, CCL2, and CCL5, driving intratumoral CD4^+^ T-cell conversion into FOXP3^+^ Tregs. PTEN loss also reduced antigen-presenting signals, including CD80, and limited cytotoxic CD8^+^ T-cell infiltration. The resulting Treg-rich, fibrotic, TGF-β-dominant microenvironment suppressed antitumor immunity and rendered NSCLC tumors resistant to anti-PD-1 therapy (Exposito et al., 2023).

#### 2.1.4 Immune resistance and evasion

In lung squamous cell carcinoma associated with COPD, TAM-derived macrophage migration inhibitory factor activates a CD74/PI3K/STAT3/PD-L1 pathway in tumor cells. Single-cell transcriptomics identified enrichment of CD74^+^ tumor cells with mixed epithelial–immune features, greater tumorigenicity, and worse prognosis. MIF–CD74 signaling increased PD-L1, suppressed CD8^+^ T-cell cytotoxicity, and promoted exhaustion. Inhibition of this axis with 4-IPP, particularly with anti-PD-1, restored CD8^+^ T-cell function and inhibited tumor growth (Wang D. et al., 2024). SOX2-OT is highly overexpressed in NSCLC, and it facilitates tumor cell proliferation, migration, and invasion, and represses cell apoptosis. Functionally, SOX2-OT acts as a ceRNA for miR-30d-5p, thus ‘sponging’ miR-30d-5p and upregulating PDK1. PDK1 then activates the mTOR pathway to increase PD-L1 expression. Functionally, this led to the promotion of immune escape by inducing CD8^+^ T-cell apoptosis and decreasing T-cell infiltration (Figure 2). Moreover, overexpression of SOX2-OT increased NSCLC tumor growth *in vivo*, and upregulated PDK1, Ki67, and PD-L1 (Chen Z. et al., 2021).

TGF-β exerts more complex, time-dependent effects on adaptive immune escape. In lung adenocarcinoma, TGF-β activated SHP1 through AKT- and Smad3-dependent phosphorylation, leading to JAK1/2 and STAT1 dephosphorylation and reduced expression of IFN-γ-responsive immune-evasion genes, including PD-L1, IDO1, HVEM, and Gal-9. Early combined TGF-β and PD-L1 inhibition improved T-cell infiltration and tumor control; however, prolonged dual blockade accelerated IFN-γ-dependent resistance through renewed interferon-stimulated gene expression, which was reversed by JAK1/2 inhibition (Ye et al., 2023) (Figure 2). More generally, PD-L1 engagement of PD-1 inhibits CD28–PI3K–AKT signaling, thereby reducing T-cell activation and IL-2 and IFN-γ production. Although this observation was demonstrated in breast cancer cell models rather than lung cancer, it supports the principle that the magnitude of tumor PD-L1 expression influences immunosuppressive capacity and responsiveness to checkpoint blockade (Parry et al., 2005).

#### 2.1.5 Tobacco smoke

In gefitinib-resistant EGFR-mutant PC9/GR and HCC827/GR cells, tobacco extract increased PD-L1, phosphorylated AKT, and mTOR signaling in a dose- and time-dependent manner. Rapamycin suppressed these effects and reduced tobacco-induced IL-6 and IL-23 transcription, indicating mTOR dependence. IL-6 was a key mediator because mTOR blockade reduced IL-6 secretion, PD-L1 induction, and macrophage chemotaxis, whereas recombinant IL-6 restored PD-L1 expression. Tobacco exposure therefore reinforces malignant behavior and immune suppression in resistant NSCLC through an mTOR/IL-6/PD-L1 pathway (Xiao et al., 2020).

#### 2.1.6 Immunosuppressive microenvironment regulation

PI3K/AKT/mTOR signaling was recently discovered to be activated by UPP1 to drive an immunosuppressive microenvironment. UPP1 promotes an immunosuppressive lung adenocarcinoma microenvironment through PI3K/AKT/mTOR activation. UPP1 overexpression increased PD-L1, suppressed CD8^+^ T-cell cytotoxicity, and enhanced TGF-β1 production. This promoted FOXP3^+^ Treg differentiation, CD8^+^ T-cell exhaustion, M2 macrophage polarization, cancer-associated fibroblast activation, and accumulation of PD-L1^+^ myeloid cells. UPP1 inhibition reversed these changes, restored CD8^+^ T-cell function, and resensitized tumors to anti-PD-L1 therapy (Li et al., 2024). RRM2 also regulates immune escape through the ANXA1/AKT/PD-L1 axis. RRM2 upregulation increased ANXA1 and AKT phosphorylation, PD-L1 expression, T-cell exhaustion, and multiple inhibitory checkpoints. Across lung adenocarcinoma cohorts, RRM2-high tumors exhibited strong IFN-γ and T-cell-inflamed signatures, increased CD8^+^ infiltration, and higher PD-1, PD-L2, CTLA-4, LAG3, and TIGIT expression. Although associated with poor prognosis without treatment, this phenotype predicted greater responsiveness to PD-1/PD-L1 blockade. RRM2 silencing reduced ANXA1/AKT activation and PD-L1, suggesting that RRM2-driven immune suppression may simultaneously create vulnerability to checkpoint inhibition (Lee et al., 2023).

#### 2.1.7 DNA damage–cGAS–STING crosstalk

PI3K/AKT signaling may also influence antitumor immunity through its interaction with the DNA-damage response and cytosolic DNA sensing. At the molecular level, AKT can directly phosphorylate human cGAS at Ser305 and suppress its enzymatic activity, whereas AKT inhibition enhances cGAS-dependent IFN-β production, identifying AKT as a negative regulator of cytosolic DNA sensing (Seo et al., 2015). AKT also participates in DNA double-strand-break repair. In A549 and H460 NSCLC cells, AKT1 depletion impaired RAD51-dependent homologous recombination and increased persistent DNA double-strand breaks following irradiation (Mueck et al., 2017). More recent evidence further connects these processes to antitumor immunity. In lung adenocarcinoma and other tumor models exposed to cisplatin, activation of the ESM1–AKT1–MDM2/p53 axis attenuated DNA damage and suppressed cGAS–STING signaling, whereas AKT inhibition reversed these effects and increased cGAS, STING and IRF3 activation together with IFN-β, CCL5, and CXCL10 expression. Increased DNA damage-associated dsDNA was also associated with greater CD8^+^ T-cell infiltration (Tan et al., 2026). Consistently, in gefitinib-resistant NSCLC, suppression of AKT/p70S6K signaling by cepharanthine occurred concurrently with activation of the STING–TBK1–IRF3 cascade and increased production of IFN-β and the T-cell-recruiting chemokines CXCL9, CXCL10, and CCL5 (Kang et al., 2026). However, a direct causal link between AKT inhibition, DNA damage, and STING activation was not established in the latter study. These findings suggest two complementary mechanisms through which PI3K/AKT pathway inhibition may facilitate innate antitumor immunity: removal of direct AKT-mediated suppression of cGAS and increased persistence of DNA damage capable of providing immunostimulatory DNA. Subsequent cGAS–STING–TBK1–IRF3 activation can promote type-I interferon and chemokine signaling and thereby favor cytotoxic T-cell recruitment and priming. Nevertheless, this relationship is context dependent, and DNA damage does not invariably result in productive cGAS–STING activation. Thus, modulation of this axis provides an additional mechanistic rationale for combining PI3K/AKT/mTOR-targeted therapies with immune checkpoint blockade.

## 3 Regulation of PI3K/AKT/mTOR pathway by PD-L1

Beyond its established role as an immune checkpoint ligand, PD-L1 mediates tumor-intrinsic signaling that promotes cancer-cell survival and proliferation, creating reciprocal crosstalk with the PI3K/AKT/mTOR pathway (Lin et al., 2024). PD-L1 overexpression increases AKT, mTOR, and S6 kinase phosphorylation, thereby enhancing glycolysis, growth, and resistance to apoptosis (Deng et al., 2019). PD-L1 may sustain PI3K activation by interacting with intracellular adaptors or modulating PTEN independently of receptor tyrosine kinases (Cretella et al., 2019). Thus, PD-L1 not only suppresses antitumor immunity but also reinforces oncogenic signaling, potentially promoting tumor aggressiveness and resistance to targeted therapies and immunotherapy (Figure 3) (Yadollahi et al., 2021).

**FIGURE 3:**
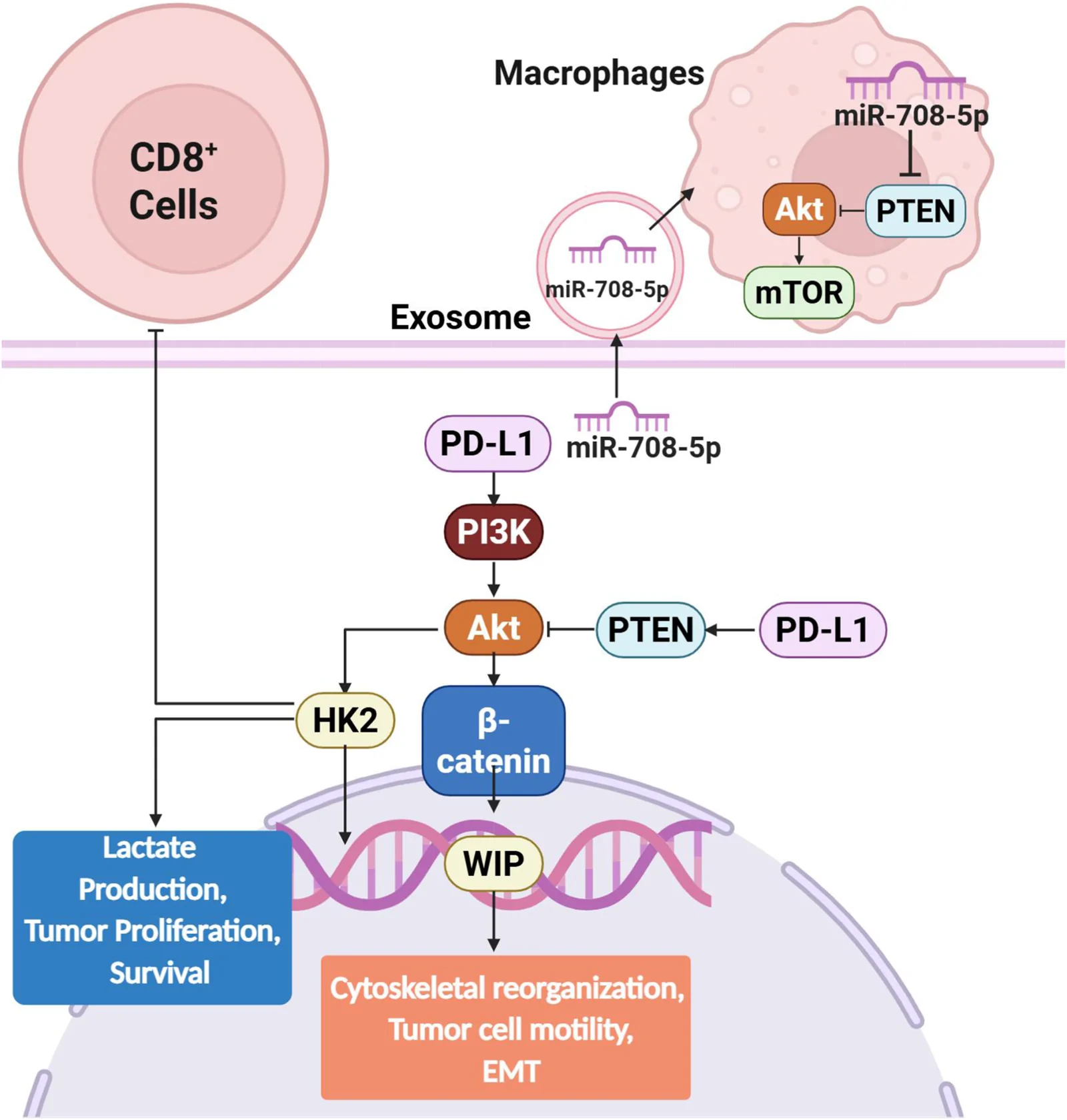
Tumor-intrinsic PD-L1 signaling has been shown to converge onto the PI3K/Akt pathway, at least in part, by suppression of PTEN (phosphatase and tensin homolog deleted on chromosome 10), and subsequent activation of mTOR, in an Akt-dependent manner to promote cell survival and proliferation in lung cancer. Akt activation, driven by PDL1-dependent integrin activation, leads to stabilization of β-catenin, its nuclear translocation, and transcriptional upregulation of WIP, which, in turn, drives cytoskeletal remodeling, EMT, and increased tumor cell motility. In addition to driving oncogenic signaling and EMT, tumor-intrinsic PD-L1 expression has also been shown to upregulate the glycolytic enzyme HK2 via PI3K/Akt, resulting in increased glycolysis and lactate production and concomitant increased tumor growth and impaired CD8^+^ T-cell effector function. In the TME, miR-708-5p-enriched LUAD-derived exosomes are uptaken by macrophages, promoting their M2 polarization, upregulation of PD-L1, and activation of the PTEN/Akt/mTOR pathway, which leads to further tumor proliferation, invasion, and immune suppression.

### 3.1 Direct PD-L1/Akt axis

PD-L1 promotes lung cancer progression through the AKT/β-catenin/WIP axis. PD-L1 overexpression enhances proliferation, migration, and invasion, whereas its silencing produces the opposite effects. Mechanistically, PD-L1 activates PI3K/AKT, leading to GSK3β inactivation, β-catenin stabilization, and its nuclear translocation. β-Catenin subsequently binds the WIP promoter and increases WIP transcription, promoting cytoskeletal remodeling, epithelial–mesenchymal transition, and tumor-cell motility. *In vivo*, PD-L1 overexpression accelerated tumor growth and increased β-catenin, WIP, and phosphorylated S6. Clinically, PD-L1 and WIP expression were positively correlated, and both were associated with poor prognosis in lung adenocarcinoma (Yu et al., 2020).

### 3.2 Exosomes

In lung adenocarcinoma, the SFRS1/miR-708-5p/PD-L1 axis mediates exosome-dependent tumor–immune crosstalk. SFRS1 promoted the selective incorporation of miR-708-5p into tumor-derived exosomes, which were internalized by macrophages and induced M2-like polarization. These macrophages expressed higher levels of IL-10, Arg-1, and PD-L1 and activated PTEN/AKT/mTOR signaling, thereby promoting tumor-cell proliferation, migration, and invasion. Macrophage PD-L1 also suppressed CD8^+^ T-cell activation and IFN-γ production. Depletion of miR-708-5p or PD-L1 reduced tumor growth, restored T-cell cytotoxicity, and reactivated PTEN signaling (Xu et al., 2025).

### 3.3 PD-L1 mediated glycolysis inhibition via PI3K/Akt pathway

Tumor-intrinsic PD-L1 signaling promotes glycolysis and immune evasion in NSCLC by upregulating hexokinase 2 (HK2) through PI3K/AKT and ERK signaling. Increased HK2 enhanced hexokinase activity, lactate production, and extracellular acidification without substantially affecting GLUT1 or PKM2, thereby reinforcing the Warburg phenotype and tumor-cell survival. In co-culture models, HK2-overexpressing NSCLC cells reduced Jurkat T-cell CD69 activation and IFN-γ secretion. Clinically, PD-L1 correlated positively with HK2, whereas HK2 was inversely associated with CD4, CD8A, IFNG, GZMB, and PRF1 expression, particularly in PD-L1-high tumors. PD-L1-high/HK2-high NSCLCs also exhibited reduced CD8^+^ T-cell infiltration and resistance to anti-PD-1 therapy, identifying the PD-L1/HK2 axis as a metabolic mechanism of immune escape (Kim et al., 2019).

## 4 PI3K/Akt/mTOR crosstalk with PD-L1: molecular view

PI3K/AKT/mTOR signaling promotes PD-L1 expression through regulators including STAT3, NF-κB, and 4E-BP1, thereby suppressing T-cell activity. Pathway inhibition can reduce PD-L1, enhance cytotoxic T-cell function, limit Treg expansion, and improve responses to PD-1 blockade in NSCLC (Quan et al., 2022). This section summarizes molecular regulators linking PI3K/AKT/mTOR signaling to PD-L1 expression and immunotherapy response (Figure 4).

**FIGURE 4:**
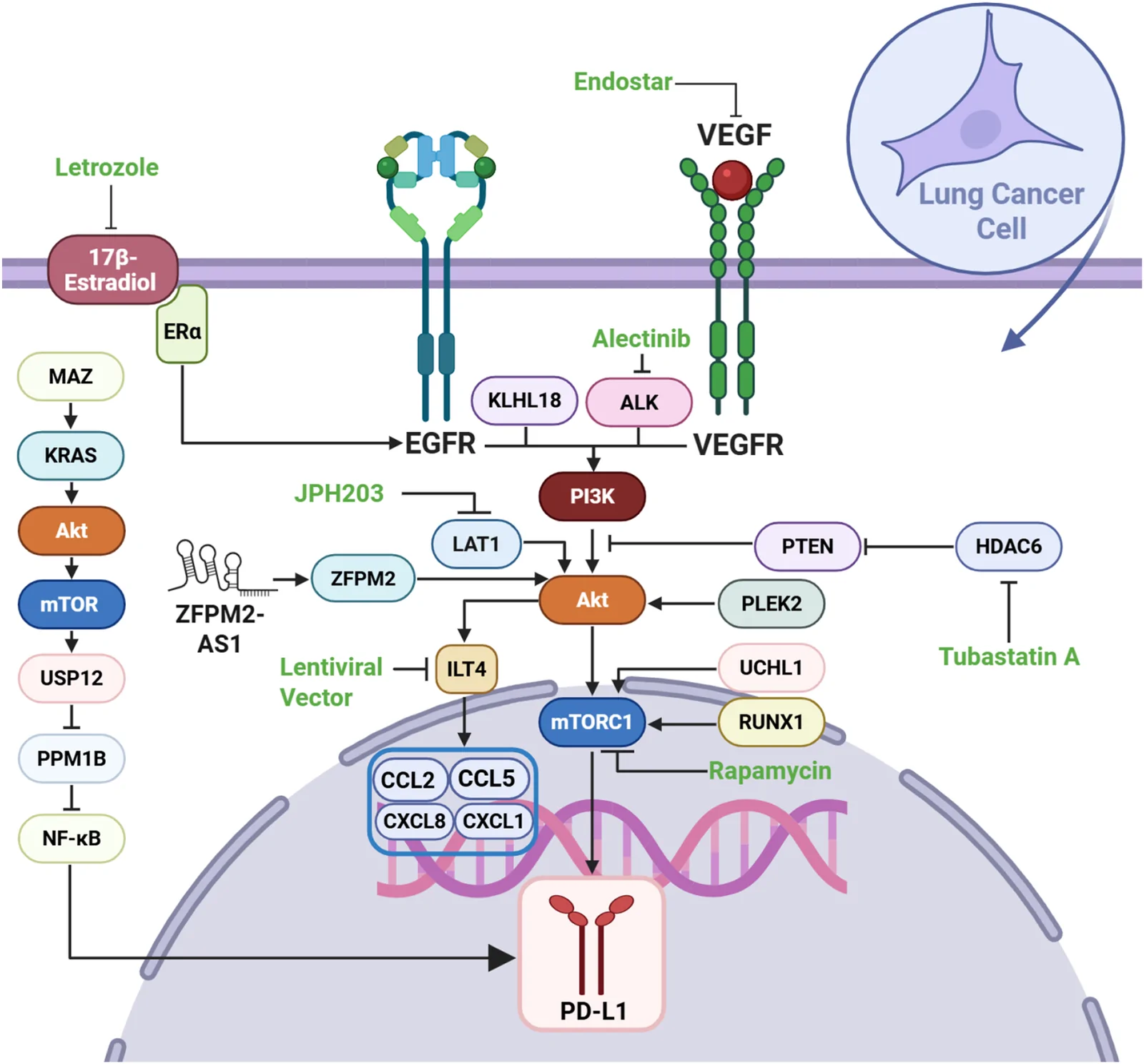
PI3K/Akt/mTOR-centered regulatory network controlling PD-L1 expression and immune escape in lung cancer. Schematic illustration summarizing how oncogenic signaling pathways converge on the PI3K/Akt/mTOR axis to regulate PD-L1 expression and modulate response to anti-PD-1 therapy in NSCLC. VEGF/VEGFR signaling promotes immune suppression and angiogenesis, which can be reversed by Endostar through inhibition of PI3K/Akt/mTOR. Multiple oncogenic drivers and modulators, including RUNX1, EGFR/ERα signaling, ALK fusion proteins, MAZ/KRAS, LAT1, PLEK2, HDAC6, ZFPM2-AS1, UCHL1, USP12, and KLHL18, either activate or restrain PI3K/Akt/mTOR signaling, thereby influencing PD-L1 transcription via downstream effectors such as NF-κB, STAT3, and mTORC1-dependent translation. Green-labeled elements denote pharmacologic agents or experimental interventions such as Endostar, alectinib, letrozole, rapamycin, JPH203, tubastatin A, and lentiviral approaches, highlighting key therapeutic nodes where pathway targeting reduces PD-L1 expression, remodels the tumor microenvironment, enhances CD8^+^ T-cell activity, and improves the efficacy of PD-1/PD-L1 checkpoint blockade.

### 4.1 VEGF

In a Lewis lung carcinoma model, VEGF inhibition with Endostar synergized with anti-PD-1 therapy by normalizing tumor vasculature and remodeling the immune microenvironment. Combined treatment reduced IL-17, TGF-β1, and MDSC infiltration while increasing IFN-γ production and CD8^+^ T-cell activation. These effects were accompanied by PI3K/AKT/mTOR inhibition, increased autophagy and apoptosis, and improved tumor control (Wu et al., 2020).

### 4.2 RUNX1

RUNX1 is upregulated in NSCLC and is associated with lymph-node metastasis, advanced stage, and poor prognosis. It binds the mTOR promoter, increasing mTOR transcription and phosphorylation, PD-L1 expression, cell-cycle regulators, and EMT markers. RUNX1 overexpression promoted proliferation and migration, whereas its silencing or rapamycin treatment reversed these effects, identifying RUNX1–mTOR signaling as a driver of NSCLC progression (Ma et al., 2023).

### 4.3 EGFR inhibition

The relationship among EGFR mutations, PI3K/AKT activation, and PD-L1 is context-dependent. In some lung adenocarcinomas, EGFR mutations correlated with increased AKT activity, but phosphorylated AKT was inversely associated with PD-L1, potentially contributing to the limited efficacy of checkpoint blockade in EGFR-mutant disease. PD-L1 may nevertheless increase after EGFR-TKI treatment, suggesting possible sensitivity to subsequent immunotherapy (Hu et al., 2020). Other data showed increased PD-L1 and AKT and reduced PI3K and PTEN expression in EGFR-mutant tumors, particularly those with exon 19 deletions. Increased AKT and reduced PTEN were also associated with lymph-node metastasis and advanced disease, supporting EGFR-dependent PD-L1 regulation through PI3K/AKT (Han and Zhang, 2019). ERα provides an additional link between EGFR signaling and immunotherapy response. In patient samples, cell lines, and immune-humanized xenografts, ERα predicted pembrolizumab response independently of sex and PD-L1. Tumor-derived estradiol and EGFR/AKT/ERK signaling activated ERα, which directly increased CD274 transcription. Letrozole reduced PD-L1 and enhanced pembrolizumab activity, increasing CD8^+^, NK, and γδ T-cell infiltration, particularly in ERα-high tumors (Anobile et al., 2023). EGFR–AKT/ERK signaling also induces ILT4, which recruits and polarizes TAMs through CCL2 and CCL5 while suppressing T-cell proliferation and cytotoxicity. ILT4 blockade restored immunity and enhanced PD-L1 inhibition in EGFR-wild-type and ligand-activated models. However, in TKI-resistant EGFR-mutant tumors, ILT4 inhibition alone, rather than its combination with PD-L1 blockade, reduced tumor progression (Chen X. et al., 2021).

### 4.4 HDAC6 inhibition

HDAC6 is overexpressed in lung adenocarcinoma and correlates with advanced stage, poor differentiation, and reduced survival. It activates PI3K/AKT/mTOR signaling to promote proliferation and EMT while supporting Treg and M2-macrophage accumulation and suppressing CD8^+^ T cells. Tubastatin A restored PTEN activity, induced apoptosis, promoted M1 polarization, and enhanced anti-PD-1 efficacy *in vivo* (Jiang et al., 2025).

### 4.5 Pleckstrin-2

Pleckstrin-2 (PLEK2) is elevated in lung adenocarcinoma tumor cells and macrophages and is associated with poor prognosis and reduced immunotherapy response. PLEK2 promoted proliferation and migration through AKT signaling, whereas its knockdown inhibited tumor growth, increased CD8^+^ T-cell infiltration, and improved anti-PD-1 efficacy *in vivo* (Yin et al., 2024).

### 4.6 MYC-associated zinc-finger protein

MYC-associated zinc-finger protein (MAZ) is upregulated in lung adenocarcinoma and predicts poor survival. It transcriptionally activates KRAS and the downstream AKT/NF-κB pathway, increasing CCL2, CXCL1, CXCL8, and Galectin-9, which promote myeloid-cell recruitment and suppress T-cell responses. MAZ silencing reduced tumor growth and immune escape *in vitro* and *in vivo* and improved the response to PD-1 blockade (Chen et al., 2024).

### 4.7 Type amino acid transporter 1

L-type amino acid transporter 1 (LAT1) LAT1 promotes cancer stemness and PD-L1 expression in NSCLC. Pemetrexed-resistant cells exhibited increased LAT1, AKT/mTOR activity, sphere formation, and stemness markers. LAT1 inhibition with RNA interference or JPH203 suppressed AKT/mTOR signaling, PD-L1, and stem-cell properties, whereas leucine supplementation reversed these effects. LAT1^+^/PD-L1^+^ tumor cells displayed the greatest stemness and were associated with poor survival in lung adenocarcinoma datasets (Liu Y.-H. et al., 2021).

### 4.8 USP12

Oncogenic AKT/mTOR signaling represses USP12 in KRAS-driven NSCLC. USP12 loss destabilizes the NF-κB inhibitory phosphatase PPM1B, resulting in NF-κB activation and increased CXCL1, CXCL8, CCL2, and CCL5. This promotes macrophage recruitment, angiogenesis, PD-L1 expression, M2-like TAM accumulation, and suppression of CD8^+^ T-cell activity, ultimately causing resistance to anti-PD-1 therapy. USP12 restoration or AKT/mTOR inhibition reversed these effects (Yang et al., 2021).

### 4.9 ZFPM2-AS1

The lncRNA ZFPM2-AS1 is overexpressed in NSCLC and is associated with larger tumors, advanced stage, and poor survival. It suppresses ZFPM2 while activating JAK2/STAT3 and AKT signaling, thereby increasing PD-L1 and promoting proliferation, migration, invasion, and immune escape. ZFPM2-AS1 silencing restored ZFPM2, inhibited STAT3 and AKT phosphorylation, and reduced PD-L1 and tumorigenicity (Wang et al., 2020a).

### 4.10 KLHL18

KLHL18 suppresses NSCLC through the PI3K/PD-L1 axis. As part of a CUL3 E3 ubiquitin-ligase complex, KLHL18 promotes ubiquitination and degradation of PI3K p85α, thereby inhibiting PI3K/AKT/mTOR signaling and PD-L1 expression. KLHL18 restoration reduced proliferation, migration, and invasion, whereas its loss promoted tumor progression and immune escape (Jiang et al., 2020).

### 4.11 UCHL1

Ubiquitin C-terminal hydrolase L1 (UCHL1) is overexpressed in NSCLC and increases PD-L1 transcription through the AKT/NF-κB p65 pathway. UCHL1 overexpression increased PD-L1, whereas genetic silencing or LDN-57444 reduced its expression. UCHL1-high tumor cells suppressed Jurkat T-cell activation and IL-2 production, an effect reversed by PD-L1 knockdown, identifying UCHL1 as a regulator of PD-L1-dependent immune escape (Mao et al., 2020).

### 4.12 ALK

PD-L1 expression is directly induced by EML4–ALK fusion oncoprotein through activation of PI3K/AKT and MEK/ERK pathways. ALK-positive NSCLC cells expressed higher PD-L1 mRNA and protein, whereas alectinib or ALK silencing reduced PD-L1. PI3K or MEK inhibition, but not STAT3 inhibition, suppressed PD-L1 in ALK-rearranged and EGFR-mutant cells. In 134 NSCLC specimens, ALK rearrangement was associated with higher PD-L1, particularly in adenocarcinomas and nonsmokers (Ota et al., 2015).

## 5 Therapeutic targeting crosstalk of PI3K/AKT/mTOR and PD-L1 in lung cancer

Targeting PI3K/AKT/mTOR signaling integrates oncogenic growth, metabolism, immune escape, and therapeutic resistance in lung cancer. Alterations such as PIK3CA mutations, PTEN loss, and EGFR or ALK activation promote tumor progression and induce PD-L1 through STAT3 and NF-κB. Conversely, PD-L1 can activate PI3K/AKT signaling, creating a feedforward loop that supports glycolysis, survival, and immune resistance (Iksen et al., 2021; Wang S. et al., 2024). Therapeutic approaches targeting this crosstalk are summarized below (Figures 5, 6; Supplementary Table S1).

**FIGURE 5:**
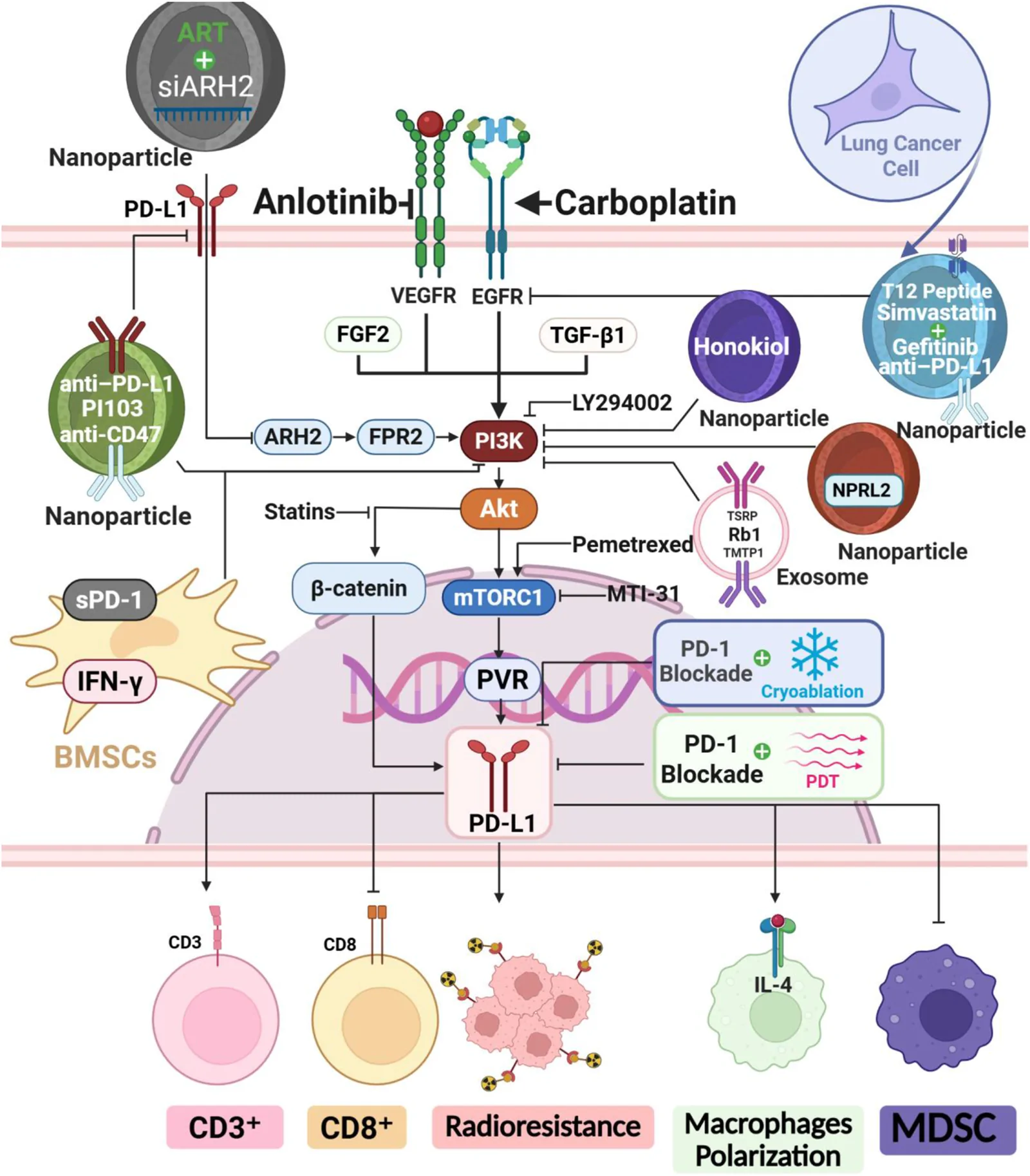
Schematic overview of the therapeutic targeting of PI3K/AKT/mTOR–PD-L1 crosstalk in lung cancer, highlighting how oncogenic signaling, chemotherapy, radiotherapy, ablation strategies, nanomedicine, exosomes, and cell-based therapies modulate tumor growth, immune evasion, and antitumor immunity.

**FIGURE 6:**
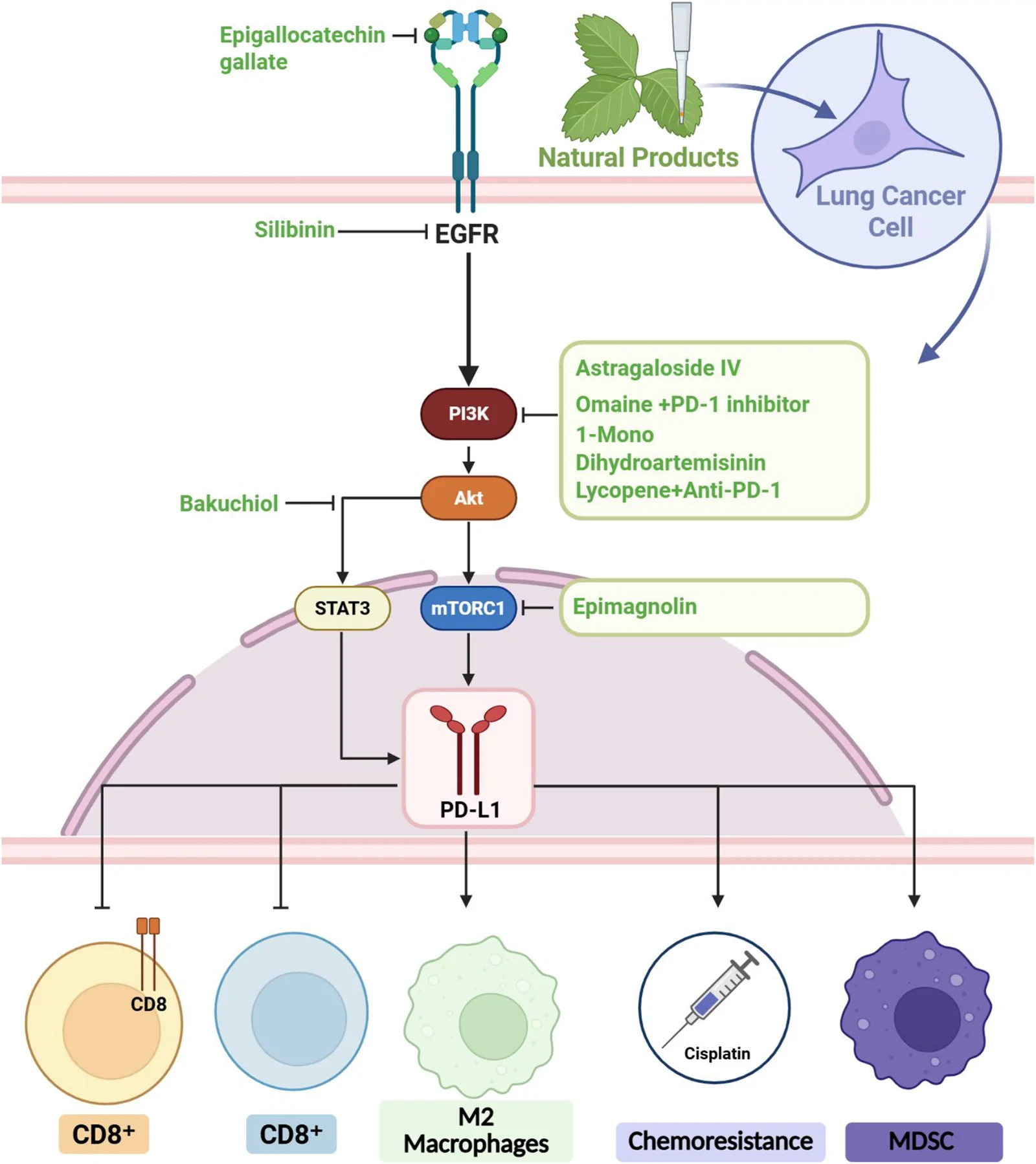
Natural products targeting the EGFR/PI3K/AKT/mTOR–PD-L1 axis in lung cancer. Multiple bioactive compounds suppress oncogenic EGFR-driven PI3K/AKT/mTOR signaling and its downstream regulators (STAT3/mTORC1), leading to reduced PD-L1 expression, restoration of CD8^+^ T-cell cytotoxicity, macrophage repolarization, attenuation of MDSCs, and reversal of chemoresistance. These multitarget actions synergize with immune checkpoint blockade (anti-PD-1/anti-CTLA-4) to remodel the tumor microenvironment and enhance antitumor efficacy.

### 5.1 Synthetic pharmaceuticals

#### 5.1.1 LY294002

A classical PI3K/AKT inhibitor, LY294002, has been shown by Zhang et al. (2017) to block immune-evasive signaling in lung cancer cells driven by TAMs. TAM-derived IFN-γ significantly increased PD-L1 surface expression and cellular invasiveness by activating the PI3K/AKT and JAK/STAT3 pathways. LY294002 ablated AKT phosphorylation and blocked PD-L1 upregulation, and partially reversed the IFN-γ driven pro-tumorigenic/immune-escape phenotype (Zhang et al., 2017). It also blocked TGF-β1/FGF2-induced PD-L1 transcription during EMT by inhibiting AKT/mTOR-dependent STAT3 and NF-κB activation in A549 and H1650 cells, but not in T790M-mutant H1975 cells. These findings implicate PI3K/AKT/mTOR signaling in EMT-associated immune evasion and EGFR-TKI resistance (Li et al., 2018).

#### 5.1.2 Carboplatin

Carboplatin dose-dependently increased PD-L1 through the EGFR/PI3K/AKT/ERK pathway in NSCLC cells. This response required poliovirus receptor, which acted upstream of PI3K/AKT. Silencing poliovirus receptor or blocking PD-L1 prevented carboplatin-induced PD-L1 expression and restored CD3^+^ T-cell proliferation, indicating that chemotherapy can simultaneously activate an immune-evasion program (Fu et al., 2023).

#### 5.1.3 Statins

Statins reduced PD-L1 transcription and protein expression in lung and melanoma cells by inhibiting the cholesterol-dependent AKT/β-catenin pathway. Suppression of AKT phosphorylation limited β-catenin activation and its binding to the CD274 promoter, whereas cholesterol supplementation reversed these effects. These findings support combining statins with PD-1/PD-L1 blockade, although part of the evidence was obtained in non-lung cancer models (Lim et al., 2021).

#### 5.1.4 Anlotinib

Anlotinib inhibited PI3K/AKT signaling and reduced PD-L1 on vascular endothelial cells, disrupting an immunosuppressive vascular barrier. This increased CD8^+^ T-cell infiltration and activation, reduced FoxP3^+^ Tregs, improved the CD8^+^/FoxP3^+^ ratio, and suppressed tumor growth. These effects provide a rationale for combining antiangiogenic therapy with immune checkpoint blockade (Liu et al., 2020).

#### 5.1.5 Pemetrexed

The antifolate pemetrexed is part of the standard treatment for non-squamous NSCLC. Pemetrexed increased membrane-bound and soluble PD-L1 through mTOR/p70S6K and STAT3 signaling, with expression persisting after drug withdrawal. Despite increasing PD-L1, pemetrexed also enhanced IFN-γ and IL-2 production and T-cell-mediated killing in co-culture models. This simultaneous inflammatory activation and adaptive PD-L1 induction may explain the clinical synergy between pemetrexed and checkpoint inhibitors (Cavazzoni et al., 2020).

#### 5.1.6 MTI-31

The dual mTORC1/mTORC2 inhibitor MTI-31 suppressed AKT and S6K phosphorylation in NSCLC models harboring EGFR, ALK, c-Met, or KRAS alterations. It promoted GSK3β-dependent proteasomal degradation of PD-L1 without changing PD-L1 mRNA, reduced T-cell apoptosis, inhibited TGF-β-induced EMT, and reversed drug resistance. MTI-31 also suppressed tumor growth, metastasis, and angiogenesis *in vivo* (Zhang et al., 2019).

### 5.2 Radiotherapy

Radiotherapy is a widely used modality for NSCLC. Irradiation increased PD-L1 through PI3K/AKT and STAT3 signaling, contributing to EMT, migration, apoptosis resistance, and radioresistance. PD-L1 silencing or blockade restored apoptosis and radiosensitivity. In mice, radiotherapy combined with anti-PD-L1 increased CD8^+^ T-cell infiltration and reduced Tregs and MDSCs, while low baseline PD-L1 was associated with better radiotherapy responses in patients (Gong et al., 2017).

### 5.3 Cryoablation

Cryoablation is a minimally invasive treatment that ablates tumor tissue through freezing, and PD-1 inhibitors are immune checkpoint blockers that reinvigorate exhausted T cells. Combining cryoablation with PD-1 blockade activated PI3K/AKT/mTOR signaling in T cells, increased CD8^+^ T-cell proliferation and IFN-γ and TNF-α secretion, reduced Tregs, inhibited tumor growth, and prolonged survival in mice. A PI3Kδ inhibitor reversed these benefits, indicating that, unlike tumor-intrinsic pathway activation, PI3K/AKT/mTOR activity in T cells is required for the antitumor effect of this combination (Liu et al., 2024).

### 5.4 Nanoparticles

Nanoparticle platforms can improve the stability, tumor accumulation, and controlled release of PI3K/AKT/mTOR-targeted agents while reducing systemic toxicity (Sanaei et al., 2022). A PD-L1-targeted, pH/ROS-responsive nanosystem co-delivering siARH2 and artesunate suppressed PI3K/AKT signaling through FPR2, inhibited M2 polarization, and induced ROS-dependent necroptosis. *In vivo*, it increased M1 macrophages and CD8^+^ T cells, reduced metastasis, and prolonged survival with limited toxicity (Cai et al., 2025). A dual-antibody nanoparticle displaying anti-PD-L1 and anti-CD47 while encapsulating the PI3K/mTOR inhibitor PI103 simultaneously blocked adaptive and innate checkpoints. It suppressed AKT/mTOR activity, enhanced macrophage phagocytosis and CD8^+^ T-cell infiltration, reduced tumor burden, and prolonged survival in syngeneic NSCLC models (Saha et al., 2024). The T12/PD-L1-Lipo system co-delivered simvastatin and gefitinib across the blood–brain barrier to PD-L1-positive brain metastases. It inhibited EGFR/AKT/ERK signaling, promoted oxidative degradation of EGFR^T790M, reversed TKI resistance, and reprogrammed macrophages from M2 to M1. The treatment reduced angiogenesis and prolonged survival with favorable biocompatibility (Yin et al., 2020). Nano-formulated honokiol (Lipo-HNK), a small molecule derived from Magnolia plant, has been reported to have potent immunomodulatory and anti-tumor synergism with PD-1 blockade in NSCLC. Liposomal honokiol inhibited PI3K/AKT signaling, M2 macrophage polarization, and macrophage PD-1/PD-L1 expression. It reduced MDSCs and M2 macrophages while enhancing cytotoxic CD8^+^ and memory CD4^+^ T-cell responses. Combined with PD-1 blockade, it produced marked regression of primary and metastatic NSCLC tumors (Cheng et al., 2024). NPRL2 gene therapy delivered via DOTAP nanoparticles restores a fundamental tumor suppressor program that can overcome anti–PD-1 resistance in KRAS/STK11-mutant NSCLC. DOTAP nanoparticle-mediated NPRL2 gene therapy restored tumor-suppressive signaling in KRAS/STK11-mutant NSCLC. NPRL2 inhibited PI3K/AKT/mTOR and MAPK pathways, induced apoptosis and cell-cycle arrest, enhanced dendritic-cell activation and CD8^+^ and NK-cell infiltration, and reduced Tregs, MDSCs, and TAMs. It synergized with pembrolizumab in responsive tumors, while overcoming resistance in KRAS/STK11-mutant tumors through partly PD-L1-independent immune reprogramming (Meraz et al., 2025).

### 5.5 Exosome delivery

Ginsenoside Rb1-loaded tumor-derived exosomes (Rb1@T-exo) functionalized with tumor-targeting peptide TMTP1 were constructed to generate a novel biomimetic nanocarrier that can effectively reverse acquired resistance to PD-1/PD-L1 immunotherapy in PI3K-mutated NSCLC. The exosomes improved drug stability and tumor targeting in PI3K-mutant NSCLC. Rb1@T-exo inhibited PI3K/AKT/mTOR and PD-L1-associated immune-evasion pathways, promoted M1 polarization, reduced M2 TAMs, and enhanced CD8^+^ T-cell proliferation, cytotoxicity, IFN-γ, and granzyme B production. Combination with nivolumab synergistically reversed immune resistance *in vitro* and *in vivo* (Jin et al., 2025). TMTP1-TSRP-engineered extracellular vesicles (TMTP1-TSRP-EVs), a biomimetic nanotherapeutic platform, were developed to reverse PIK3CA mutation–driven immune resistance and bone metastasis in NSCLC. They suppressed PI3K/AKT/mTOR signaling and PD-L1, restored cytotoxic CD8^+^ T-cell activity, promoted M1 polarization, and reduced tumor burden and bone lesions. Combination with nivolumab reversed primary checkpoint-inhibitor resistance, partly through an S6K/RNF168-dependent mechanism (Liu et al., 2025).

### 5.6 Photodynamic therapy

Mechanistically, photodynamic therapy (PDT) combined with anti-PD-1 immunotherapy exhibited a robust synergetic antitumor effect in NSCLC by activating cytotoxic immune response and inhibiting oncogenic survival signals. Ce6-based photodynamic therapy combined with anti-PD-1 produced synergistic tumor inhibition in Lewis lung carcinoma models. The treatment increased effector T-cell infiltration and the CD8^+^/FoxP3^+^ ratio while suppressing EGFR, ERK, PI3K, and AKT phosphorylation. ROS-mediated tumor killing and pathway inhibition therefore cooperated with checkpoint blockade to restore antitumor immunity (Gong et al., 2024).

### 5.7 Mesenchymal stem cells

Bone marrow mesenchymal stem cells engineered to co-express IFN-γ and soluble PD-1 inhibited proliferation, migration, and invasion and promoted apoptosis in A549 and Lewis lung cancer cells. *In vivo*, they reduced tumor burden and tissue injury, increased intratumoral CD8^+^ T cells, decreased Tregs, and suppressed phosphorylated PI3K/AKT and tumor PD-L1. These findings support engineered mesenchymal stem cells as a potential dual-delivery platform for immune activation and pathway inhibition (Xie et al., 2025).

### 5.8 Natural products: preclinical evidence and translational limitations

Natural products modulate PI3K/AKT/mTOR signaling through diverse mechanisms that affect tumor survival, proliferation, metastasis, angiogenesis, and epithelial–mesenchymal transition. As multitarget agents, they may induce apoptosis, autophagy, and cell-cycle arrest while showing relatively favorable biocompatibility and potential to overcome resistance through simultaneous regulation of parallel pathways (Figure 6) (Iksen et al., 2021). Among the more mechanistically defined compounds, tubeimoside-1 (TBM-1) directly binds the FRB domain of mTOR and inhibits mTORC1, promoting TFEB nuclear translocation and lysosome biogenesis and thereby facilitating lysosomal degradation of PD-L1. In experimental tumor models, this was associated with increased CD8^+^ T-cell activity, reduced Tregs and MDSCs, and enhanced checkpoint-directed therapy (Liu X. et al., 2021). Epimagnolin similarly represents a pathway-focused mechanistic lead because it was reported to bind the ATP-binding pocket of mTOR and preferentially suppress mTORC2/AKT signaling, resulting in reduced AKT/GSK3β/p70S6K activity and inhibition of EGF-driven transformation (Yoo et al., 2019). However, the latter study did not establish PD-L1 modulation or checkpoint-immunotherapy sensitization, illustrating the distinction between direct pathway engagement and demonstrated regulation of the immune-checkpoint axis. Several other compounds have shown PD-L1-modulatory or immunomodulatory activity predominantly in cellular and animal models. Bakuchiol suppressed IFN-γ-induced PD-L1 together with AKT and STAT3 phosphorylation and enhanced the antitumor effect of PD-1 blockade in preclinical models (Lin et al., 2023). Dihydroartemisinin reduced PD-L1 and inhibited TGF-β, PI3K/AKT, STAT3, and NF-κB signaling in NSCLC and radioresistant models, while improving the response to radiotherapy and favorably altering immune-cell composition *in vivo* (Zhang et al., 2020). Lycopene enhanced anti-PD-1 activity in Lewis lung carcinoma while increasing CD8^+^ T-cell infiltration and suppressing AKT phosphorylation and IFN-γ-induced PD-L1 (Jiang et al., 2019). Similarly, EGCG inhibited IFN-γ- and EGF-induced PD-L1 through JAK2/STAT1 and EGFR/AKT signaling and improved T-cell activity in experimental systems (Rawangkan et al., 2018). Astragaloside IV also enhanced anti-PD-1 activity and altered macrophage polarization in Lewis lung carcinoma while suppressing PI3K/AKT and ERK signaling (Wu et al., 2024). Collectively, these studies provide proof of principle that naturally derived molecules can influence oncogenic signaling and immune regulation, but they do not yet establish clinically achievable modulation of the PI3K/AKT/mTOR–PD-L1 axis. The strength of mechanistic evidence is particularly variable for complex botanical preparations. For example, Semen coicis reduced PI3K/AKT/mTOR phosphorylation and IFN-γ-induced PD-L1 in A549 cells, but the proposed interactions of individual constituents with PIK3CA, AKT2, and mTOR were based partly on computational predictions rather than direct biochemical target-engagement experiments ((Fu et al., 2024). Likewise, Huoxue Yiqi Recipe-2 reduced PI3K, AKT, and PD-L1 expression and altered macrophage polarization in experimental lung-cancer models (Teng et al., 2020), but its multicomponent composition makes assignment of these effects to a specific molecular target difficult. Such formulations also introduce additional translational challenges related to batch composition, active-component identification, exposure, and reproducibility. Some natural compounds have a greater degree of human exposure than their preclinical immune-checkpoint literature alone might suggest. Ginsenoside Rg3, for example, has been evaluated clinically as an adjunct to chemotherapy or EGFR-targeted therapy in NSCLC (Jiang et al., 2017), while Coix-seed-derived formulations have also undergone clinical investigation (Fu et al., 2024). Silibinin has similarly been explored in human lung-cancer-related settings (Rugamba et al., 2021). Nevertheless, these clinical observations should be distinguished from validation of the mechanisms discussed here: current evidence does not establish that clinical benefit from these agents is mediated by pharmacologically verified inhibition of PI3K/AKT/mTOR, reduction of tumor PD-L1, or enhancement of PD-1/PD-L1 blockade. Thus, existing human experience can support further investigation but cannot yet be considered clinical confirmation of the proposed checkpoint-related mechanisms. Therefore, natural products provide useful mechanistic leads for targeting signaling and immune pathways in lung cancer, but most evidence remains preclinical. Their effects on PI3K/AKT/mTOR signaling or PD-L1 in experimental models should not be considered evidence of clinical efficacy. Translation will require standardized formulations, rigorous PK/PD and target-engagement studies, and prospective clinical trials. Accordingly, these agents should currently be viewed as investigational adjuncts rather than established alternatives to pathway inhibitors or immune checkpoint blockade.

## 6 Conclusion

The evidence reviewed here indicates that the PI3K/AKT/mTOR–PD-L1 network functions as a central integrator of lung cancer progression and immune escape through both tumor-cell-intrinsic and microenvironmental mechanisms. Within tumor cells, PI3K/AKT/mTOR signaling regulates PD-L1 at multiple levels, including transcription, translation, intracellular trafficking, and protein turnover. These effects are highly context dependent and vary according to oncogenic-driver status, cytokine exposure, and previous treatment. Conversely, PD-L1 should not be viewed solely as an immune checkpoint ligand, because tumor-intrinsic PD-L1 signaling can feed back into the PI3K/AKT/mTOR pathway, partly through PTEN-associated mechanisms, thereby reinforcing AKT/mTOR activity, glycolytic metabolism, cell survival, and resistance to stress. This reciprocal circuit may explain why immune escape often accompanies aggressive tumor behavior and why single-agent therapies frequently fail to produce durable responses.

Within the tumor microenvironment, this signaling network coordinates immune suppression across both myeloid and lymphoid compartments and thereby contributes to resistance to immunotherapy. PI3K/AKT/mTOR-dependent tumor programs alter the production of paracrine mediators that recruit and reprogram macrophages, neutrophils, and T-cell subsets. These signals promote immunosuppressive macrophage polarization, cytotoxic CD8^+^ T-cell exhaustion, and accumulation of FOXP3^+^ regulatory T cells. Together, these changes weaken antigen-specific antitumor immunity and establish conditions that favor either primary or acquired resistance to PD-1/PD-L1 blockade. Regulation of this network involves numerous upstream factors, including EGFR and ALK signaling, VEGF-dependent angiogenic pathways, transcriptional and epigenetic regulators, ubiquitination and deubiquitination systems, metabolic transporters, nutrient-sensing pathways, non-coding RNAs, and extracellular vesicles. Despite the diversity of these inputs, they frequently converge on similar downstream outcomes, including enhanced survival under stress, EMT-associated migration and invasion, metabolic changes that impair immune-cell function, and reinforcement of immunosuppressive niches.

From a therapeutic perspective, disruption of PI3K/AKT/mTOR–PD-L1 signaling provides a common rationale for combining conventional and emerging treatment modalities. Chemotherapy and radiotherapy can induce PD-L1 as an adaptive response; therefore, inhibiting PI3K/AKT/mTOR signaling may counteract immune escape while simultaneously weakening the survival and repair pathways that maintain resistant tumor-cell populations. Small-molecule inhibitors, ablation-based immune-priming approaches, nanoparticles, biomimetic delivery systems, engineered extracellular vesicles, and cell-based therapies have all been investigated as strategies to suppress PD-L1, modulate PI3K/AKT/mTOR signaling, and remodel the tumor microenvironment. Across these platforms, recurring effects include reduced PD-L1 expression or activity, increased CD8^+^ T-cell infiltration and activation, decreased accumulation of Tregs and MDSCs, macrophage repolarization toward inflammatory phenotypes, attenuation of EMT and metastatic behavior, and metabolic changes that support antitumor immunity. The natural products discussed in this review often converge on comparable mechanisms through multitarget regulation of PI3K/AKT/mTOR-associated phosphorylation and transcriptional programs. Many also enhance sensitivity to checkpoint blockade; however, clinical translation will require more rigorous mechanistic validation, formulation standardization, and pharmacological characterization.

Several important limitations and unresolved questions remain. Much of the available mechanistic evidence is derived from selected cell lines and animal models that do not adequately reproduce interpatient heterogeneity, prior treatment exposure, or the spatial determinants of immune infiltration and exclusion. PD-L1 regulation downstream of mTOR is also highly context dependent, with potentially opposing effects on translation, trafficking, and protein stability. These differences may limit the generalizability of individual findings and influence the optimal sequence of combination therapies. In addition, the systemic toxicity of pathway inhibitors, their possible on-target effects on immune cells, and the dosing schedules required to suppress tumor signaling without compromising T-cell competence remain incompletely understood. Reliable predictive biomarkers for selecting patients most likely to benefit from combined targeting of PI3K/AKT/mTOR and PD-1/PD-L1 are still lacking, and comparative evidence remains uneven across inhibitor classes, delivery systems, and natural-product formulations.

Future investigations should prioritize biomarker-guided treatment selection and the mechanism-based design of rational combinations. Genomic alterations such as PTEN loss and PIK3CA mutations, the broader oncogenic-driver context, the dominant mechanisms controlling PD-L1, and the immune phenotype of the tumor are likely to influence treatment choice and sequencing. Spatial and single-cell profiling, longitudinal tumor sampling, and functional immune assays may help identify compensatory pathways that sustain resistance and define the most effective therapeutic window. Tumor-selective delivery approaches, including nanoparticles, engineered vesicles, and other cell-targeting platforms, may improve drug accumulation in tumor cells or specific microenvironmental compartments, increase the therapeutic index, and facilitate safer combination of PI3K/AKT/mTOR modulation with immune checkpoint blockade. For natural products, key priorities include confirmation of direct molecular targets, standardized and reproducible formulations, pharmacokinetic evaluation, and controlled studies of combination therapy.
