# Supplementary material for: PI3K/AKT/mTOR signaling at the immune checkpoint interface in lung cancer: molecular mechanisms, Tumor–Immune remodeling, therapeutic resistance, and emerging treatment strategies
Source: Front Cell Dev Biol. 2026 Sep 25;14:1952310. doi: 10.3389/fcell.2026.1952310 (PMC13650427; doi:10.3389/fcell.2026.1952310)
Supplement: Supplementary file 1 [file Table1.docx]

**Supplementary Table 1**- Therapeutic methods and drugs targeting the crosstalk between PD-L1 and PI3K/Akt/mTOR in lung cancer.

| **Therapeutic agent** | **Mode of treatment** | **Experimental cell models** | **Experimental design** | **Effect on PD-L1 expression or regulation** | **Effect on PI3K/Akt/mTOR pathway activity** | **Functional outcome on tumor cell behavior** | **Ref.** |
| --- | --- | --- | --- | --- | --- | --- | --- |
| **Chemotherapy** | | | | | | | |
| **LY294002** | Pharmacologic inhibitor of the PI3K/AKT signaling pathway used to block IFN-γ-induced signaling in lung cancer cells | Human lung adenocarcinoma cell line A549 | In vitro study; A549 cells stimulated with recombinant IFN-γ or tumor-associated macrophage (TAM) supernatant, then treated with LY294002 to assess pathway and PD-L1 changes | Inhibited IFN-γ-induced PD-L1 upregulation on A549 cell surfaces; reduced PD-L1 expression compared to untreated IFN-γ group | Suppressed phosphorylation of AKT, confirming inhibition of the PI3K/AKT pathway; blocked downstream activation leading to PD-L1 transcription | Reduced invasive potential of lung cancer cells and partially reversed immune-evasive phenotype, suggesting LY294002 may counteract TAM/IFN-γ–driven tumor progression | (Zhang et al., 2017) |
| **Carboplatin** | Platinum-based chemotherapeutic agent; tested alone or with pathway inhibitors (Gefitinib, LY294002, PD98059) and PVR knockdown | Human NSCLC cell lines H292, A549, and H1299 | In vitro: dose-dependent carboplatin exposure; mRNA-seq; siRNA-mediated PVR knockdown; CD3⁺ T-cell coculture assays; validation in clinical NSCLC samples | Strongly induced PD-L1 mRNA and protein expression in a dose-dependent manner; effect abolished by PVR knockdown or pathway inhibitors | Activated EGFR, PI3K/AKT, and ERK signaling (↑p-PI3K, ↑p-AKT, ↑p-ERK); inhibitors (Gefitinib, LY294002, PD98059) suppressed PVR and PD-L1 induction, confirming pathway dependence | Suppressed CD3⁺ T-cell proliferation via PD-L1; PVR silencing or PD-L1 blockade restored T-cell activity; indicates carboplatin-induced immune evasion through PVR–PI3K/AKT–PD-L1 axis | (Fu et al., 2023) |
| **Statins (Simvastatin, Atorvastatin, Lovastatin, Fluvastatin)** | Cholesterol-lowering HMG-CoA reductase inhibitors used to explore their immunomodulatory and anti-tumor roles; tested alone and in combination with cholesterol supplementation or AKT inhibition | Human lung cancer (Calu-1, NCI-H460, NCI-H358) and melanoma (A375, A2058) cell lines | In vitro: dose- and time-dependent statin treatment; qRT-PCR, Western blot, ChIP-qPCR, and AKT inhibition assays; β-catenin promoter binding analysis | Significantly reduced PD-L1 mRNA and protein expression in a dose-dependent manner; reversed by cholesterol supplementation (25-HC); suppression occurred at the transcriptional level via inhibition of β-catenin promoter occupancy | Decreased phosphorylation of AKT (Ser473) and β-catenin (Ser552); ERK and GSK3β unaffected; indicates statins inhibit AKT–β-catenin axis independently of prenylation | Reduced immune-evasive potential of tumor cells by downregulating PD-L1; provides a mechanistic basis for synergy between statins and PD-1/PD-L1 checkpoint blockade in NSCLC and melanoma | (Lim et al., 2021) |
| **Anlotinib** | Multi-target tyrosine kinase inhibitor (VEGFR, FGFR, PDGFR) with antiangiogenic and immunomodulatory effects | Endothelial cells: HUVECs, bEnd.3, mouse retinal microvascular endothelial cells (MRMECs); Tumor models: B16 melanoma, MC38 colon adenocarcinoma | In vitro: endothelial cells exposed to tumor-conditioned medium, hypoxia, or VEGFA ± anlotinib; AKT activator (SC79) and inhibitor (LY294002) used to confirm pathway involvement. In vivo: C57BL/6 mice bearing B16 or MC38 tumors treated with anlotinib, bevacizumab, or combination | Downregulated PD-L1 expression on vascular endothelial cells (VECs) in vitro and in vivo; reversed by AKT activation (SC79); counteracted bevacizumab-induced PD-L1 upregulation | Inhibited PI3K/AKT phosphorylation in endothelial cells; VEGFR/PDGFR/FGFR blockade by anlotinib reduced p-AKT and downstream PD-L1 expression; ERK signaling unaffected | Remodeled tumor immune microenvironment: increased CD8⁺/FoxP3⁺ T-cell ratio, reduced FoxP3⁺ Tregs, enhanced CD8⁺ T-cell activity (↑IFN-γ, ↑Granzyme B), decreased IL-10/IL-4 secretion, and suppressed tumor growth; efficacy dependent on CD8⁺ T cells | (Liu et al., 2020) |
| **Pemetrexed** | Antifolate chemotherapeutic; part of platinum doublet and immunochemotherapy combinations | NSCLC adenocarcinoma cell lines (A549, Calu-6, H292, H322); activated human PBMCs | In vitro drug treatment ± pathway inhibitors (RAD001 for mTOR, C188-9 for STAT3); co-culture with T cells; evaluation of PD-L1, cytokine production, and cytotoxicity | Upregulated membrane and soluble PD-L1 expression via mTOR/P70S6K and STAT3 activation; sustained PD-L1 even post-drug removal | Activated mTOR and STAT3 pathways; inhibition of either reduced PD-L1 induction; IFN-γ/IL-2 from T cells further reinforced PD-L1 expression | Enhanced T-cell activation and cytokine release (↑IFN-γ, ↑IL-2, ↓TGF-β); potentiated anti-PD-L1 (atezolizumab) cytotoxicity; promoted pro-inflammatory, immune-active tumor microenvironment | (Cavazzoni et al., 2020) |
| **MTI-31** | Dual mTORC1/mTORC2 inhibitor (mTOR kinase inhibitor); targeted therapy for mTOR-dependent, TKI-resistant NSCLC | EGFR-mutant (H1975, PC9), ALK-mutant (H2228), c-Met-driven (H1993), KRAS-mutant (A549) NSCLC cells; Jurkat and primary human CD3⁺ T cells; xenograft and metastasis models | In vitro drug treatments ± pathway inhibitors or genetic mTORC1/2 disruption; in vivo subcutaneous, intracranial, and metastatic NSCLC models; T-cell coculture assays | Downregulated PD-L1 via mTORC2/AKT/GSK3β-mediated proteasomal degradation; inhibition of both mTOR complexes markedly reduced PD-L1 protein levels | Blocked phosphorylation of AKT (Ser473) and S6K, suppressing both mTORC1 and mTORC2 activity; reduced downstream EMT and survival signaling | Inhibited tumor proliferation, EMT, migration, and metastasis; restored chemosensitivity; decreased T-cell apoptosis and enhanced antitumor immunity through PD-L1 suppression | (Zhang et al., 2019) |
| **Radiotherapy** | | | | | | | |
| **Radiotherapy ± anti–PD-L1 antibody** | Conventional fractionated radiotherapy (2 Gy × 3 fractions, total 6 Gy) ± anti–PD-L1 antibody | A549, PC9, H520, A549/X (radioresistant), PC9/R (TKI-resistant), Lewis lung carcinoma (LLC) models; NSCLC patient cohort (n = 76) | In vitro irradiation; si-PD-L1 knockdown; in vivo mouse models treated with anti–PD-L1; retrospective analysis of chemoradiotherapy patients | Radiation markedly upregulated PD-L1 in radiosensitive NSCLC lines and patient tumors; PD-L1 knockdown or blockade restored radiosensitivity and induced apoptosis | Activated PI3K/AKT and STAT3 pathways after irradiation; si-PD-L1 reduced p-AKT and p-STAT3, linking pathway activation to PD-L1 induction | PD-L1 promoted EMT, migration, and radioresistance (↑vimentin, Snail, N-cadherin, ↓E-cadherin); anti–PD-L1 + RT synergistically enhanced CD8⁺ T-cell infiltration, reduced Tregs/MDSCs, and suppressed tumor growth | (Gong et al., 2017) |
| **Nanoparticles** | | | | | | | |
| **pH/ROS-responsive nanosystem co-delivering siARH2 and artesunate (ART)** | Multifunctional nanoplatform integrating PD-L1-targeted peptides, pH/ROS-sensitive release, and dual immune activation via siARH2 (immune reprogramming) and ART (necroptosis induction) | LUAD cell lines (A549, LLC), macrophages (THP-1, MH-S), and tumor-bearing mouse models | In vitro co-culture assays (macrophage polarization, ROS generation, necroptosis); in vivo imaging, IHC, and survival analysis | Targeted delivery via PD-L1-binding peptides enhanced tumor accumulation and uptake; indirectly reduced PD-L1-positive immunosuppressive cells in TME | ARH2 silencing upregulated FPR2 and inhibited PI3K/AKT phosphorylation, reversing M2 macrophage polarization; ART-induced ROS amplified this inhibition | Induced necroptosis and ROS in tumor cells, decreased M2 macrophages, increased M1 and CD8⁺ T-cell infiltration, suppressed metastasis, and prolonged survival; showed excellent biosafety | (Cai et al., 2025) |
| **Anti–CD47–PD-L1 antibody–conjugated nanoparticle loaded with PI103 (ADN)** | Dual immune checkpoint blockade (PD-L1/CD47) combined with PI3K/mTOR inhibition via targeted drug delivery; nanocarrier mimicking ADCs but with higher antibody density | Human LUAD (A549), murine LLC and KLN-205 models (C57BL/6, DBA/2 mice), macrophage (RAW264.7) co-culture | In vitro receptor binding, internalization, cytotoxicity, phagocytosis, and pathway inhibition assays; in vivo antitumor efficacy and immunophenotyping | Blocked PD-L1 signaling and increased phagocytic clearance through dual checkpoint inhibition; enabled selective targeting of PD-L1⁺ tumor cells | PI103 cargo inhibited phosphorylation of AKT and mTOR, suppressing PI3K/Akt/mTOR signaling in tumor cells | Enhanced phagocytosis, increased CD8⁺ T-cell infiltration, suppressed tumor growth, reduced hemolysis/toxicity vs monotherapies; synergistic innate + adaptive immune activation | (Saha et al., 2024) |
| **Dual-targeting liposome modified with transferrin receptor (T12) peptide and anti–PD-L1 nanobody, co-delivering simvastatin (SV) and gefitinib (Gef)** | Brain-targeted nanotherapy designed for BBB penetration, TAM repolarization, and reversal of EGFRT790M-mediated drug resistance | H1975 NSCLC (EGFRT790M⁺) intracranial xenografts, BCECs, HUVECs, TAM (M1/M2) co-culture | In vitro BBB-penetration and co-culture assays; in vivo imaging, survival, and histology in brain metastasis mouse models | Targeted TAMs and tumor vasculature via PD-L1 binding, enhancing uptake in PD-L1⁺ and TfR⁺ brain tumors; indirectly reduced PD-L1⁺ immunosuppressive macrophages | Inhibited EGFR/Akt/Erk signaling, increased ROS, downregulated MsrA and GPX4, promoted oxidative degradation of EGFRT790M, and reversed PI3K/Akt-driven resistance | Repolarized M2→M1 macrophages, induced apoptosis, suppressed angiogenesis, prolonged survival, reversed TKI resistance, and showed excellent BBB permeability and biosafety | (Yin et al., 2020) |
| **NPRL2 (Nitrogen Permease Regulator–Like 2) gene therapy using cationic DOTAP nanoparticles** | Tumor suppressor gene restoration to induce DNA damage, apoptosis, and activate immune responses in anti–PD-1–resistant KRAS/STK11-mutant NSCLC | Humanized and syngeneic mouse models bearing A549 (KRAS/STK11mut, PD-1R), H1299 (KRASwt, PD-1S), and LLC2 (KRASmut, PD-1R) tumors | In vitro NPRL2 transfection assays, apoptosis/colony formation, phospho-signaling analyses, and in vivo humanized mouse lung metastasis and subcutaneous tumor models ± pembrolizumab | Upregulated PD-L1 in PD-1–responsive tumors (H1299) enabling synergy with pembrolizumab; unchanged PD-L1 in PD-1–resistant KRAS/STK11mut tumors, suggesting PD-L1-independent immune reactivation | Downregulated PI3K/AKT/mTOR and MAPK phosphorylation, reduced phospho-S6, 4E-BP1, PRAS40, GSK-3β; NPRL2 interacts with Rag GTPases to inhibit mTORC1 signaling | Induced apoptosis, DNA damage, DC activation, CD8⁺ T-cell expansion, reduced Tregs, MDSCs, and TAMs, restored antigen presentation, and overcame anti–PD-1 resistance in KRAS/STK11mut NSCLC | (Meraz et al., 2025) |
| **Tubeimoside-1 (TBM-1) (triterpenoid saponin from *Bolbostemma paniculatum*)** | Small-molecule immune modulator that directly binds and inhibits mTORC1, promoting TFEB-dependent lysosomal degradation of PD-L1 and restoring antitumor immunity | NSCLC cell lines (A549, H157, H1299, H460), melanoma (A375, B16), Lewis lung carcinoma (LLC) xenografts | In vitro PD-1/PD-L1 binding, lysosomal activity (LysoTracker, NAG assays), siRNA knockdown (TFEB/mTOR), CETSA, SPR, molecular docking; in vivo C57BL/6 tumor models ± anti-CTLA-4 antibody | Significantly decreased PD-L1 abundance via post-translational lysosomal degradation (autophagy-independent); reduced PD-1 binding and restored T-cell cytotoxicity | Directly bound and inactivated mTORC1, reduced phosphorylation of p70S6K, 4EBP1, and TFEB Ser211, leading to TFEB nuclear translocation and lysosome biogenesis | Enhanced CD8⁺ T-cell infiltration, increased IFN-γ and granzyme-B⁺ TILs, decreased Tregs and MDSCs, suppressed tumor growth, and synergized with anti-CTLA-4; no systemic toxicity observed | (Liu et al., 2021a) |
| **Astragaloside IV (AS-IV) (active saponin from *Astragalus membranaceus*) ± anti-PD-1 antibody** | Combination immunotherapy enhancing anti-PD-1 efficacy via TME reprogramming and inhibition of pro-survival signaling | C57BL/6J mice bearing Lewis lung carcinoma (LLC) | In vivo treatment (AS-IV 40 mg/kg Q3D; anti-mPD-1 10 mg/kg BIW); IHC, IF, TUNEL, WB, and macrophage/T-cell marker analyses | PD-L1 levels not directly assayed, but functional suppression inferred from enhanced anti-PD-1 activity and increased T-cell activation | Significant inhibition of PI3K, Akt, and ERK phosphorylation in tumor tissues, indicating blockade of pro-survival and immunosuppressive signaling | Reduced tumor volume and Ki-67, increased apoptosis (TUNEL, cleaved caspase-3), shifted TAMs from M2→M1 phenotype (↑CD86, ↓CD206), enhanced CD3⁺CD69⁺ T-cell activation, and synergistic tumor regression without toxicity | (Wu et al., 2024) |
| **Liposomal Honokiol (Lipo-HNK) — nanoliposomal formulation of a natural phenolic compound from *Magnolia officinalis*** | Nanodrug + anti-PD-1 combination therapy; modulates macrophage polarization and immune checkpoint signaling | Human NSCLC (H460, A549) and murine LL2 cell lines; C57BL/6 mice (subcutaneous + metastatic models) | Cell viability, apoptosis, colony formation, flow cytometry, IF, RNA-seq, WB for PI3K/AKT; macrophage-depletion and T-cell activation assays | ↓ PD-1 and PD-L1 mRNA and protein levels in macrophages; reversed IL-4–induced PD-L1 upregulation via PI3K inhibition | ↓ PI3Kα/β and p-AKT (Ser473); inhibition of IL-4–induced signaling blocked M2 polarization; PI3K agonist restored M2 traits | Inhibited tumor growth, reduced MDSC and M2 macrophages, increased CD8⁺ T-cell activation (CD69⁺/Granzyme B⁺), upregulated memory CD4⁺ T cells, and synergized with anti-PD-1 therapy for tumor regression without toxicity | (Cheng et al., 2024) |
| **Extracellular Vesicles** | | | | | | | |
| **TMTP1-TSRP-engineered CSC-derived EVs (TMTP1-TSRP-EVs)** | Tumor-targeting extracellular vesicles carrying transformable specific-responsive peptide (TSRP); nanocarrier designed to inhibit PI3K/Akt/mTOR signaling and reverse ICI resistance | PIK3CA-mutant A549 and H1703 NSCLC cells; CD8⁺ T cell co-culture; NOD-SCID xenograft and bone metastasis models | In vitro (WB, qPCR, CCK-8, Transwell, TUNEL, flow cytometry); in vivo (IVIS imaging, micro-CT, IHC, bone metastasis suppression, Nivolumab combination) | TSRP and TMTP1-TSRP-EVs markedly downregulated PD-L1 protein in PIK3CA-mutant NSCLC and restored anti–PD-1 efficacy; increased CD8⁺ T-cell infiltration | Strongly suppressed PI3K, AKT, and mTOR phosphorylation; reduced downstream S6K and RNF168 expression even under AKT activation (SC79) | Inhibited proliferation, migration, and bone metastasis; enhanced apoptosis; increased M1 macrophage and CD8⁺ T-cell infiltration; reduced M2 macrophages; reversed primary ICI resistance and improved anti–PD-1 (Nivolumab) responsiveness without systemic toxicity | (Liu et al., 2025) |
| **Ginsenoside Rb1-loaded, peptide-enhanced tumor exosomes (Rb1@T-exo)** | Exosome-based nanocarrier system delivering ginsenoside Rb1 via tumor-targeting peptide TMTP1; designed to overcome acquired immunotherapy resistance in PI3K-mutant NSCLC | PI3K-mutated A549, 344SQ_R (anti-PD-1-resistant) cell lines; xenograft, bone metastasis, and PD-1-resistant NSCLC mouse models | In vitro (CCK-8, WB, flow cytometry, RNA-seq, qPCR, ELISA) and in vivo (fluorescence imaging, tumor growth, IHC, bone metastasis, immune profiling) | Transcriptomic and KEGG analyses showed suppression of PD-L1 expression pathway and re-sensitization to anti-PD-1 therapy | Strongly inhibited PI3K, p-AKT, and p-mTOR; downregulated mTOR- and hypoxia-related DEGs; restored T-cell signaling | Suppressed tumor growth and bone metastasis; promoted M1 macrophage polarization (↑CD86, iNOS, TNF-α); reduced M2 TAMs (↓CD206, IL-10, Arg-1); enhanced CD8⁺ T-cell proliferation, IFN-γ/Granzyme B production, and cytotoxicity; synergized with Nivolumab to reverse immune resistance and restore anti-tumor immunity | (Jin et al., 2025) |
| **Photodynamic Therapy** | | | | | | | |
| **Photodynamic Therapy (PDT) combined with Anti-PD-1 antibody** | Physical phototherapy using Ce6 photosensitizer (660 nm, 300 mW/cm²) combined with systemic anti-PD-1 immunotherapy; aims to synergize local ROS-induced cytotoxicity with checkpoint inhibition | Lewis lung carcinoma (LLC)–bearing C57BL/6J mice | In vivo: Ce6-PDT every 3 days ± anti-PD-1 injections; RNA-seq, qRT-PCR, WB, IF, immune infiltration (CD8⁺, Foxp3⁺) | Indirect PD-L1 regulation via suppression of EGFR/ERK/PI3K/Akt pathway and enhanced CD8⁺ infiltration; reduced immunosuppressive signaling | Decreased phosphorylation of EGFR, ERK1/2, PI3K, and Akt; downregulation of ADAM12; upregulation of ALAS2 and ITGA10 associated with increased immune activation | Inhibited tumor growth and proliferation; increased CD8⁺ T-cell infiltration; decreased Foxp3⁺ Treg cells; improved body weight and survival; promoted immune infiltration and antitumor cytokine response; synergistic tumor regression compared with monotherapies | (Gong et al., 2024) |
| **Bone marrow-derived mesenchymal stem cells** | | | | | | | |
| **IFN-γ- and sPD-1-overexpressing bone marrow-derived mesenchymal stem cells (BMSCs)** | Gene-engineered cell-based immunotherapy delivering IFN-γ and soluble PD-1 to enhance anti-tumor immune response and inhibit immune evasion | Human A549 and murine Lewis lung adenocarcinoma cells; C57BL/6J tumor-bearing mice | In vitro (co-culture proliferation, migration, invasion, and senescence assays); in vivo (subcutaneous tumor model, TUNEL, flow cytometry, IHC, qPCR, WB) | Downregulated PD-L1 mRNA and protein levels in tumor tissue; enhanced PD-1/PD-L1 blockade efficacy; reduced immune suppression | Decreased PI3K and AKT mRNA/protein expression and phosphorylation; inhibited downstream survival signaling | Suppressed tumor proliferation, migration, and invasion; induced apoptosis (↑Bax, ↓Bcl-2) and senescence; reduced Treg levels; increased CD8⁺ T-cell infiltration; improved lung pathology; enhanced overall immune activation and tumor regression | (Xie et al., 2025) |
| **Cryoablation** | | | | | | | |
| **Cryoablation + PD-1 inhibitor** | Combined local ablation with immune checkpoint blockade | Lewis lung carcinoma (LLC) cells; C57BL/6N mice bearing bilateral tumors | In vivo mouse model of lung adenocarcinoma; cryoablation of one tumor followed by systemic PD-1 inhibitor injections | Cryoablation alone increased PD-L1 and PD-1 expression, contributing to immune evasion; PD-1 blockade reversed this effect by preventing T-cell exhaustion | Combination therapy markedly activated the PI3K/AKT/mTOR pathway in CD8⁺ T cells (↑p-PI3K, ↑p-AKT, ↑p-mTOR); inhibition with PI3Kδ-IN-15 reversed this activation | Synergistic inhibition of tumor growth, enhanced CD8⁺ T-cell proliferation and cytokine release (↑IFN-γ, ↑TNF-α), reduced Tregs, prolonged survival, and induced tumor-specific immune memory | (Liu et al., 2024) |
| **Natural products** | | | | | | | |
| **Z-guggulsterone (Z-GS)** | Natural sterol isomer; FXR antagonist with anti-tumor potential | A549, H1975 (human NSCLC); LLC (mouse lung carcinoma) | In vitro (cell viability, apoptosis, PD-L1, FXR knockdown/overexpression); in vivo (LLC xenograft in mice) | Upregulated PD-L1 mRNA, protein, and promoter activity in a dose- and time-dependent manner, partly via FXR inhibition | Activated Akt (Ser473) and Erk1/2 (Thr202/Tyr204) phosphorylation; inhibitors (MK2206, PD0325901) attenuated PD-L1 induction | Suppressed cell viability, induced G0/G1 cell cycle arrest, and reduced tumor growth in vivo despite PD-L1 upregulation | (Tian et al., 2021) |
| **Semen coicis (Coix seed extract) combined with PD-1 inhibitor** | Natural compound–based adjuvant immunotherapy enhancing checkpoint blockade efficacy through inhibition of PI3K/AKT/mTOR signaling | A549 NSCLC cell line; human PBMC co-culture | In vitro MTT, RT-PCR, WB, ELISA, and flow cytometry assays | Downregulated PD-L1 expression on A549 cells induced by IFN-γ stimulation; synergistic suppression with PD-1 inhibitor | Decreased phosphorylation of PI3K, AKT, and mTOR; reduced PI3K, AKT, and mTOR mRNA levels | Enhanced tumor cell apoptosis and growth inhibition; decreased cytokines IFN-γ, IL-6, TNF-α; alleviated immunosuppression and improved T-cell-mediated response | (Fu et al., 2024) |
| **Bakuchiol (BAK)** | Herb-derived prenylated phenolic monoterpene; immune-modulatory and PD-L1 inhibitory agent | Lewis lung carcinoma (LLC) cells; C57BL/6 mice bearing LLC xenografts | In vitro (qPCR, WB, IHC); in vivo (oral gavage 5–40 mg/kg, 15 days) ± PD-1 blockade | Significantly downregulated PD-L1 mRNA and protein levels in tumors and IFN-γ–stimulated LLC cells; reversed PD-L1 upregulation induced by IFN-γ | Inhibited phosphorylation of AKT and STAT3, both at baseline and after IFN-γ stimulation | Suppressed tumor growth; enhanced CD8⁺ T cells and M1 macrophages, reduced Tregs and M2 macrophages; shifted TME toward pro-inflammatory state (↑IL-1β, IL-2, IFN-γ, TNF-α); synergized with PD-1 blockade to prolong survival | (Lin et al., 2023) |
| **Silibinin (natural flavonolignan from *Silybum marianum*)** | Natural anticancer compound; EGFR-binding inhibitor targeting PD-L1 via PI3K/AKT and JAK2/STAT5 suppression | A549, H292, H460 (NSCLC cell lines); HUVECs for angiogenesis assays | In vitro assays (MTT, flow cytometry, WB, qPCR, ChIP, invasion, migration, and tumorsphere formation) | Significantly downregulated PD-L1 at transcriptional and protein levels; disrupted STAT5 binding to PD-L1 promoter (confirmed by ChIP assay) | Inhibited EGFR phosphorylation and its downstream JAK2/STAT5 and PI3K/AKT pathways; decreased p-EGFR, p-JAK2, p-STAT5, and p-AKT without altering total protein levels | Induced G0/G1 arrest, apoptosis, inhibited angiogenesis (↓VEGF), migration, invasion (↓MMP2/9), and tumorsphere formation (↓SOX2, NANOG, OCT4); suppressed CSC stemness and tumor progression | (Rugamba et al., 2021) |
| **Dihydroartemisinin (DHA)** | Natural sesquiterpene lactone; radiosensitizer combined with radiotherapy | A549, PC9, A549/X, PC9/X (radioresistant NSCLC); Lewis lung carcinoma (LLC) xenograft model | In vitro (cell viability, apoptosis, PD-L1 knockdown, signaling analysis); in vivo (combined DHA + radiotherapy in mice) | Downregulated PD-L1 expression induced by radiotherapy; blocked immune escape and reversed PD-L1-associated radiation resistance | Inhibited TGF-β, PI3K/Akt, STAT3, and NF-κB activation; enhanced TRIM21 and modulated EMT-related proteins | Enhanced radiosensitivity, induced apoptosis (↑Bim, cleaved caspases-3/8; ↓Bcl-2), inhibited EMT, reduced MDSCs/iTregs, increased CD8⁺ T-cell infiltration, and synergistically suppressed tumor growth | (Zhang et al., 2020) |
| **Lycopene (carotenoid from tomatoes and red fruits)** | Natural compound; adjuvant to anti-PD-1 therapy enhancing immune activation via IFN pathway and PI3K/AKT modulation | Lewis lung carcinoma (LLC) cells; C57BL/6 mice with LLC tumors | In vivo (anti-PD-1 ± lycopene); in vitro (IFNγ-stimulated LLC cells; RT-PCR, Western blot, bisulfite sequencing) | Suppressed IFNγ-induced PD-L1 transcription and protein expression via AKT inhibition; no change in basal PD-L1 in vivo | Promoted JAK2 activation and AKT dephosphorylation, enhanced IFN signaling (↑IRF1/7, CXCL9, CXCL10); reduced DNMT3a and demethylated IRF1/7 promoters | Synergized with anti-PD-1 to reduce tumor growth, increase CD8⁺ IFNγ⁺ T cells, elevate IL-2/IFNγ and decrease IL-4/IL-10; enhanced apoptosis and tumor immune clearance | (Jiang et al., 2019) |
| **(−)-Epigallocatechin gallate (EGCG) (main catechin in green tea)** | Natural polyphenol; acts as an alternative immune checkpoint inhibitor and EGFR/JAK pathway modulator | A549, H1299, Lu99 (human NSCLC); F10-OVA (melanoma) co-culture; A/J mice (NNK-induced lung tumors) | In vitro (IFN-γ and EGF stimulation assays; STAT1/Akt phosphorylation; co-culture with CD3⁺ T cells); in vivo (oral green tea extract 0.3% in drinking water for 16 weeks) | Inhibited IFN-γ- and EGF-induced PD-L1 mRNA and protein by 40–80%; reduced PD-L1⁺ tumor cells in vivo by ~70% | Suppressed p-STAT1 (↓85%) and p-Akt (↓43%); blocked JAK2/STAT1 and EGFR/Akt pathways; mimicked JAK2 inhibitor TG-101348; unaffected by PI3K inhibitor wortmannin on PD-L1 | Decreased tumor number (−37%) and PD-L1⁺ cells (−70%) in NNK-treated mice; restored IL-2 in tumor-specific CD3⁺ T cells; enhanced T-cell proliferation; partially reversed immune suppression | (Rawangkan et al., 2018) |
| **Ginsenoside Rg3 (steroidal saponin from *Panax ginseng*)** | Natural compound; adjuvant to chemotherapy targeting PD-L1-mediated immune escape and cisplatin resistance | A549 (parental) and A549/DDP (cisplatin-resistant) human NSCLC cells; CD8⁺ T-cell co-culture | In vitro (MTT assay, apoptosis, PD-L1/Akt/NF-κB Western blot, T-cell cytotoxicity assay) | Decreased PD-L1 expression in A549/DDP cells; reversed PD-L1 overexpression induced by cisplatin resistance | Suppressed phosphorylation of Akt (Ser473) and NF-κB p65 (Ser536); blocked PI3K/Akt- and NF-κB-mediated PD-L1 upregulation | Enhanced cisplatin cytotoxicity and apoptosis, resensitized resistant cells, and restored CD8⁺ T-cell cytotoxicity against tumor cells | (Jiang et al., 2017) |
| **Huoxue Yiqi Recipe-2 (HYR-2) — herbal formula derived from *Ze Qi Decoction* (Salvia miltiorrhiza, Panax ginseng, Glycyrrhiza uralensis)** | Traditional Chinese herbal formulation targeting tumor proliferation and immune evasion; modulates PD-L1 and gut microbiota | H1975, A549, H1299, 95-D lung cancer cells; LLC-bearing C57BL/6 mice | In vitro (MTT, colony formation, qRT-PCR, WB) and in vivo (21-day oral administration, tumor growth, gut 16S rRNA sequencing) | Downregulated PD-L1 mRNA and protein in H1975 cells and LLC tumors; enhanced M1 macrophage polarization | Inhibited EGFR/PI3K/Akt phosphorylation; suppressed downstream signaling driving PD-L1 transcription | Inhibited proliferation and colony formation of lung cancer cells; reduced tumor volume and weight in vivo; induced M1/M2 repolarization; increased intestinal Akkermansia muciniphila abundance, synergistically enhancing PD-L1-related immunotherapy potential | (Teng et al., 2020) |
